# Applying conservation reserve design strategies to define ecosystem monitoring priorities

**DOI:** 10.1002/ece3.8344

**Published:** 2021-11-11

**Authors:** Irene Martín‐Forés, Greg R. Guerin, Samantha E. M. Munroe, Ben Sparrow

**Affiliations:** ^1^ School of Biological Sciences The University of Adelaide Adelaide SA Australia; ^2^ Terrestrial Ecosystem Research Network (TERN) University of Adelaide Adelaide SA Australia

**Keywords:** biodiversity, diversity partitioning, endemism, monitoring network, optimization, prioritization, species turnover

## Abstract

In an era of unprecedented ecological upheaval, monitoring ecosystem change at large spatial scales and over long‐time frames is an essential endeavor of effective environmental management and conservation. However, economic limitations often preclude revisiting entire monitoring networks at high frequency. We aimed here to develop a prioritization strategy for monitoring networks to select a subset of existing sites that meets the principles of complementarity and representativeness of the whole ecological reality, and maximizes ecological complementarity (species accumulation) and the spatial and environmental representativeness. We applied two well‐known approaches for conservation design, the “minimum set” and the “maximal coverage” problems, using a suite of alpha and beta biodiversity metrics. We created a novel function for the R environment that performs biodiversity metric comparisons and site prioritization on a plot‐by‐plot basis. We tested our procedures using plot data provided by the Terrestrial Ecosystem Research Network (TERN) AusPlots, an Australian long‐term monitoring network of 774 vegetation and soil monitoring plots. We selected 250 plots and 80% of the total species recorded as targets for the maximal coverage and minimum set problems, respectively. We compared the subsets selected by the different biodiversity metrics in terms of complementarity and spatial and environmental representativeness. We found that prioritization based on species turnover (i.e., iterative selection of the most dissimilar plot to a cumulative sample in terms of species replacement) maximized ecological complementarity and spatial representativeness, while also providing high environmental coverage. Species richness was an unreliable metric for spatial representation. Selection based on range‐rarity‐richness was balanced in terms of complementarity and representativeness, whereas its richness‐corrected implementation failed to capture ecological and environmental variation. Prioritization based on species turnover is desirable to cover the maximum variability of the whole network. Synthesis and applications: Our results inform monitoring design and conservation priorities, which can benefit by considering the turnover component of beta diversity in addition to univariate metrics. Our tool is computationally efficient, free, and can be readily applied to any species versus sites dataset, facilitating rapid decision‐making.

## Graphical abstract

Here, we propose an easy and straightforward prioritization strategy for monitoring networks to select a subset of plots that maximizes not only ecological complementarity but also environmental and spatial representativeness. We applied two well‐known approaches for conservation reserve design, the “minimum set” and the “maximal coverage” problems using a suite of alpha and beta biodiversity metrics and compare the performance of such biodiversity metrics according to the subsets of plots selected by them complementarity and representation of the whole network. The subset of monitoring sites based on species turnover was the one that best represented the ecological reality of the whole network in terms of species accumulated and environmental and spatial coverage.

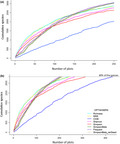

## 1 INTRODUCTION

Ecological monitoring is a prerequisite for successful environmental policy and decision‐making, and the development of effective management and conservation programs (Haase et al., 2018; Jeffers, 1989; Jones, 2011; Lovett et al., 2007; Parr et al., 2003; Sparrow, Edwards, et al., 2020; Spellerberg, 2005; Vos et al., 2000; Wolfe et al., 1987). Over the past two decades, monitoring programs have been developed at large scales to incorporate broader landscape processes (Parr et al., 2003; Sparrow, Edwards, et al., 2020; Yoccoz et al., 2001). There are now several examples of these comprehensive ecosystem observation networks established at continental scale, including the pan‐European Integrated Carbon Observation System (ICOS), the National Ecological Observatory Network (NEON; USA), the Global Ecosystems Monitoring (GEM) network across the tropics (Malhi et al., 2021), and the Terrestrial Ecosystem Research Network (TERN) in Australia (Cleverly et al., 2019).

Such monitoring programs require large financial investments to provide standardized surveying training, fieldwork organization, sample preservation and storage, as well as data curation, access, and promotion (Kang et al., 2016). Therefore, the sampling breadth and frequency of ecosystem monitoring programs are limited by resource constraints (Lovett et al., 2007). Consequently, monitoring resources need to be carefully prioritized, including in the determination of efficient site revisit schedules (James et al., 1999; Kang et al., 2016).

Here, we aimed to design a straightforward prioritization strategy for large‐scale monitoring networks by comparing a suite of commonly used alpha and beta biodiversity metrics and identifying which one best optimizes the selection of a subset of plots that maximizes the number of species accumulated, while ensuring environmental and spatial representativeness. To do so, we developed a free and user‐friendly tool for the R environment to perform an optimization process applying the maximal coverage problem, *optim_species* function (included in the *ausplotsR* package; Guerin, Saleeba, et al., 2020; see Appendix S1 for R code details).

When resources are limited, “conservation prioritization”‐style strategies can be implemented to select an optimal subset of sites for monitoring. This includes ensuring high information content (i.e., the largest possible set of species), and meeting the principles of complementarity and representativeness (Bennett et al., 2014; Guerin et al., 2020; Guerin et al., 2020; Justus & Sarkar, 2002; Kirkpatrick, 1983; Margules & Pressey, 2000). Indeed, several analyses have sought to find optimal subsets of larger ecological samples for efficiency of sampling effort (Bennett et al., 2014; Dowd et al., 2014). For example, Pesch et al. (2008) reported that over 300 sites could be removed from an existing monitoring network for moss while remaining ecologically, environmentally, and spatially representative across many ecoregions.

Monitored sites should also constitute a spatially and environmentally representative subset of locations to ensure ecological and statistical validity (Cullen, 1990; Sparrow, Foulkes, et al., 2020; Vos et al., 2000). A cost‐effective resampling strategy needs to define a subset of priority sites to be revisited and overcome the existing resource‐limited trade‐offs between effective spatial and temporal monitoring (Hewitt & Thrush, 2007). While species richness has commonly been used to prioritize sites for monitoring or conservation, it may not be efficient for this purpose, nor ensure complementarity (Gotelli & Colwell, 2001; Hillebrand et al., 2018; Justus & Sarkar, 2002; Kirkpatrick, 1983).

The “minimum set” problem and the “maximal coverage” problem are two common approaches to prioritize conservation reserves aiming to maximize conservation benefits at minimum costs (McIntosh et al., 2017). The minimum set problem is based on ecological constraints; it identifies a set of plots that meets certain conservation targets (typically species) within the fewest possible number of sites (Margules & Pressey, 2000). In contrast, the maximal coverage problem is based on economic constrains and consists of maximizing the number of species in a given number of sites (Alagador & Cerdeira, 2020).

While heuristic algorithms (e.g., Marxan, Zonation or prioritizr) are effective as optimizers for both minimum set and maximal coverage problems, they are often based on species distribution models (Amorim et al., 2014; Carvalho, 2016) and can require complex analytical decisions or be computationally expensive (Ball et al., 2009; Pressey et al., 1996). Alternative approaches based on simpler optimization strategies have been employed; for example based on range‐rarity richness (RRR; i.e., richness weighted by the inverse of the number of sites in which each species is present; Albuquerque & Beier, 2015; Guerin & Lowe, 2015; Veach et al., 2017) or on endemism and threatened species (Smith et al., 2008) for nature reserve design in California and a trans‐frontier conservation area in Southern Africa, respectively.

The species turnover component (i.e., species replacement) of beta diversity (Baselga, 2010) has been proposed as one of the most robust biodiversity metrics to detect ecological changes over time, as it reflects compositional change within plant communities and is a strong indicator of how those communities respond to global change (Hillebrand et al., 2018). Yet, the use of species turnover to define conservation priorities and complementarity is still scarce (but see Socolar et al., 2016). No studies have compared its performance to other biodiversity metrics with regards not only to ecological complementarity but also to spatial and environmental representativeness. Given the different approaches employed in the literature, it is worth investigating how the different biodiversity metrics perform when applying to site prioritization in a continental ecological monitoring program.

Monitoring optimization approaches have been previously developed; however, their complexity often precludes them from considering more than one dimension; thus, either they are focused on ensuring species targeting (e.g., Morán‐Ordóñez et al., 2018), or on maximizing environmental representativeness (e.g., O’Hare et al., 2020).

We compared the utility of biodiversity metrics in selecting subsets of sites using a methodology applied to TERN AusPlots, an Australian long‐term monitoring network of ecosystem surveillance and monitoring sites distributed at continental scale (*sensu* Eyre et al., 2011; Sparrow, Edwards, et al., 2020). We aimed to select a subset of sites that optimize the complementarity and representativeness of the whole network, and to compare the efficiency of various metrics to do this. We applied both the minimum set and maximal coverage approaches to design a revisiting strategy for the collection of AusPlots. Specifically, we aimed to identify which biodiversity metrics could be most efficiently used to achieve an optimal revisiting strategy that maximizes ecological complementarity (i.e., the number of species accumulated) when imposing (i) an ecological constraint (i.e., minimum set problem—consisting on covering 80% of the total number of species recorded in the dataset) and (ii) an economic constraint (i.e., maximal coverage problem—consisting on selecting a subset of 250 plots). For the results of the maximal coverage problem approach (i.e., those obtained with the *optim_species* function), we subsequently compared the environmental and spatial representativeness of the subsets of sites selected by each of those biodiversity metrics as optimizers to determine, which is the preferred option to meet our complementarity and representativeness principles.

## 2 METHODS

### 2.1 Case study dataset

Our study uses AusPlots, a plot network originally designed via environmental stratification (Guerin, Williams, Leitch, et al., 2020; Guerin, Williams, Sparrow, et al., 2020) that has been systemically surveyed over 10 years by TERN's Ecosystem Surveillance Program, a component of Australia's land ecosystem observatory (Cleverly et al., 2019). We used species presence and cover data from 774 one‐hectare plots to compare biodiversity metrics and test our site prioritization approach. The plots are established in homogenous areas of terrestrial vegetation to take quantitative measurements of vegetation and soil characterization (Guerin et al., 2017). In each plot, vegetation structure and composition are recorded using the point‐intercept module (Sparrow, Foulkes, et al., 2020). Parallel transects (10 × 100 m long) are laid out in a 5 × 5 grid pattern, spaced 20 m apart. Species identity, cover and growth form are recorded at each 1 m point along each transect, resulting in 1010 survey points per plot. Data for each plot are available and freely accessible within the AusPlots database, and were extracted from the database using *ausplotsR* (v1.2; Guerin, Saleeba, et al., 2020; Munroe et al., 2021; TERN, 2020).

Some of the sites included in the dataset were revisited (i.e., 99 sites had been revisited, 73 of them twice, and 26 three times) and different sets of species were recorded. Where repeat visits occurred, each was treated as a sample (hereafter, we refer to each visit as a “plot” for simplicity).

### 2.2 Biodiversity metrics

Using the function *optim_species* from the ausplotsR package, we compared a selection of often‐used biodiversity metrics to be employed as optimizers to define monitoring priorities. The biodiversity metrics included univariate metrics: (i) species richness, (ii) range‐rarity richness (RRR; Guerin & Lowe, 2015), (iii) corrected range‐rarity richness (CRRR; Crisp et al., 2001; Guerin & Lowe, 2015), (iv) Shannon‐Wiener diversity index (Shannon; Shannon & Weaver, 1949), and (v) Simpson diversity index (Simpson; Simpson, 1949). Species turnover‐based metrics used included: i) pairwise Simpson dissimilarity (Simpson_Beta; Baselga, 2010) and ii) the most frequently selected plots after repeating pairwise Simpson dissimilarity selection over 1,000 iterations starting with different seed plots (“Frequent”). The optim_species function calculates the alpha metrics for each plot, and ranks them, selecting the n top ones (e.g., 250 plots). For the beta metrics, the function chooses a seed plot, calculates the dissimilarity between the seed and all other plots, and chooses the most dissimilar one. Afterward, the most dissimilar one gets merged with the previous one and the process is repeated, finding the next most different to the cumulative sample (i.e., it is an iterative process where each step depends on the former one. See Table 1, Appendix S2 and Guerin, Saleeba, et al. (2020)) for further details of both, the optimizers and the function.

**TABLE 1:** Optimizer's description

Optimizer ID	Optimizer name	Description	Special utilisation/Best used
Richness	Species richness	Count of the number of species present in a given site	Identify biodiversity hotspots
RRR	Range‐rarity richness	Inverse of the number of sites in which a species occurs. RRR = $\sum_{1}^{n}1/c_{i}$, where $c_{i}$ is the number of map grid cells occupied by species *i* and *n* is the number of species	When the goal is to identify areas of high biodiversity and biological uniqueness
CRRR	Corrected range‐rarity richness	Range rarity richness (RRR) divided by species richness. CRRR = $(\sum_{1}^{n}1/c_{i}$)/*n*, where $c_{i}$ is the number of map grid cells occupied by species *i* and *n* is the number of species	When the goal is to identify centers of endemism or highlight range‐restricted species
Shannon	Shannon‐Wiener diversity index	Combines species richness and the evenness or equitability by computing the species' relative abundances. H': $‐\sum_{i=1}^{S}p_{i}\log_{n}p_{i}$, where S is the species richness and *p_i_* is the relative abundance of the species	It assumes that all species are represented in a sample and that they are randomly sampled
Simpson	Simpson diversity index	Combines species richness and the evenness or equitability by computing the species' relative abundances *D* = 1 − Σ$p_{i}$ *^2^*, where $p_{i}$is the proportional abundance of species *i*	It is the complement of Simpson's original dominance index, and represents the probability that two randomly chosen individuals belong to different species
Simpson_Beta	Pairwise Simpson dissimilarity index	It is based on diversity partitioning, which separates species replacement (i.e., turnover) from species loss (i.e., nestedness). The Simpson dissimilarity corresponds to the turnover component of the Sorensen dissimilarity. Considering two sites, *β*sim = min(*b*, *c*)/(*a* + min(*b*, *c*)), where *a* is the number of species present in both sites, *b* is the number of species present in the first site, but not in the second, and *c* is the number of species present in the second site, but not in the first.	It is used to maximize species turnover
Frequent		The most frequent plots selected over 1,000 iterations with a randomized starting seed using the pairwise Simpson dissimilarity index	
Simpson_Random		The pairwise Simpson dissimilarity index with a randomized starting seed iterated 1,000 times	

### 2.3 Data analyses

#### 2.3.1 Multisite beta diversity

We carried out all statistical analyses in R (R Core Team, 2020). To check to what degree biodiversity differences between plots were due to species replacement or species loss we computed multiple‐site Sorenson dissimilarities in beta diversity (βsor) accounting for both the spatial turnover (βsim) and the nestedness (βnes) components of beta diversity (βsor = βsim + βnes; Baselga et al., 2018; Koleff et al., 2003).

#### 2.3.2 Conservation reserve design applied to optimize monitoring strategies

We applied the maximum coverage and the minimum set problems to optimize monitoring site selection to prioritize sites to revisit. For both the minimum set problem and the maximal coverage problem, we performed the analyses by selecting individual plots. Subsequently, we applied the maximal coverage problem to spatial clusters of plots to consider a more realistic and feasible scenario because it is unlikely a field team would go to a remote area to only sample one plot, for example.

We developed the R function *optim_species* as part of this study which builds on functionalities from the *vegan* (Oksanen et al., 2019) and *betapart* (Baselga et al., 2018) packages. The optimization analysis is captured in this function which can be accessed in *ausplotsR* (Guerin, Saleeba, et al., 2020). The function is thus free and easily accessible and can be run on any similar dataset (see R code as well as another example in the Appendices S1 and S3 for details). Hence, we performed the analyses employing the *optim_species* function, using as data input the species versus sites matrix in terms of presence/absence, except for Shannon and Simpson, for which we used the matrix including percent cover values.

##### The maximal coverage problem

To address the maximal coverage problem, we set to 250 the number of plots to be selected for future revisits and monitoring. We decided on 250 plots within the AusPlots monitoring network because it is a feasible number of plots to revisit on a three‐to‐five‐year cycle.

##### The minimum set problem

To address the minimum set problem, we elucidated how many plots we would need to be revisited using each optimizer to account for at least 80% of the overall species richness (2,822 species). The minimum set problem was analyzed employing the same optimizers described for the maximal coverage problem (Table 1).

#### 2.3.3 Spatial coverage representativeness

To compare spatial coverage representativeness of the plots selected by different optimizers, we computed the Clark and Evans aggregation index (Clark & Evans, 1954) for the spatial point patterns obtained with each of the optimizers using spatstat (Baddeley et al., 2015). We applied the cumulative distribution function *cdf* without edge correction because of corresponding to the mean value of nearest neighbor distance distribution function G(r) from a point pattern within an arbitrary shape. The Clark‐Evans test values show whether a spatial point pattern distribution is clustered (*R* < 1), or ordered or regular (*R* > 1). We also mapped the location of the 250 selected plots obtained from the maximal coverage problem to visually support the differences in spatial representativeness when applying each of the biodiversity optimizers.

#### 2.3.4 Environmental coverage representativeness

We compared sets of optimized plots for their climatic representativeness across Australia. We extracted data for 25 climatic variables from Harwood et al. (2016) (Appendix S4). We assessed plant species composition data from field plots in the order they were selected by the different optimizers, treating successive plots as additions to a cumulative sample of environmental and ecological space. We computed Euclidean distances for environmental variables with the function *vegdist* from the vegan package to assess the environmental representativeness of the subsets of plots selected by different optimizers. We implemented the *betadisper* function to analyze multivariate homogeneity of dispersions (distance to group centroid in principal coordinates space) of the cumulative samples (Anderson et al., 2006) for the different optimizers. We plotted the cumulative mean of environmental variation against the subsets of plots selected and visually compared the representativeness when using each of the biodiversity metrics as optimizers. Finally, we conducted a permutation test for homogeneity of multivariate dispersions with 999 permutations to explore pairwise comparisons between optimizers with regards to environmental coverage.

#### 2.3.5 Monitoring strategy optimization considering logistics

##### Spatial clustering

To make the optimization more realistic in terms of field work feasibility, we clustered the 774 plots by geographic distance using a modified version of the *CalcDists* function (https://gist.github.com/sckott/931445) in which we estimated the distances among plots with the *distCosine* function from the geosphere package (Hijmans, 2019). The final number of clusters was 68, with an average number of eleven plots (nine sites) within each of them. The number of sites within each cluster ranged from three to 24 (Appendix S5).

We aggregated the species presence/absence data of species in the plots comprising each cluster. For the cover data, we calculated the Shannon and Simpson indices per plot, and then calculated the average value of the index for all the plots. We set to 20 the number of clusters to be selected via the same optimization process. We then compared the species accumulation in the top 20 clusters when employing each of the biodiversity metrics.

## 3 RESULTS

### 3.1 Multisite beta diversity across Australia

A total of 3528 species were recorded across all of the sampled plots (*n* = 774 plots). The multisite Sorenson dissimilarity index was 0.998, the species turnover component (i.e., Simpson dissimilarity) corresponded to 0.997, while the nestedness component was only 0.001, indicating a very high rate of species replacement across the distributed plot network.

### 3.2 Conservation reserve design applied to optimize monitoring strategies

**FIGURE 1:**
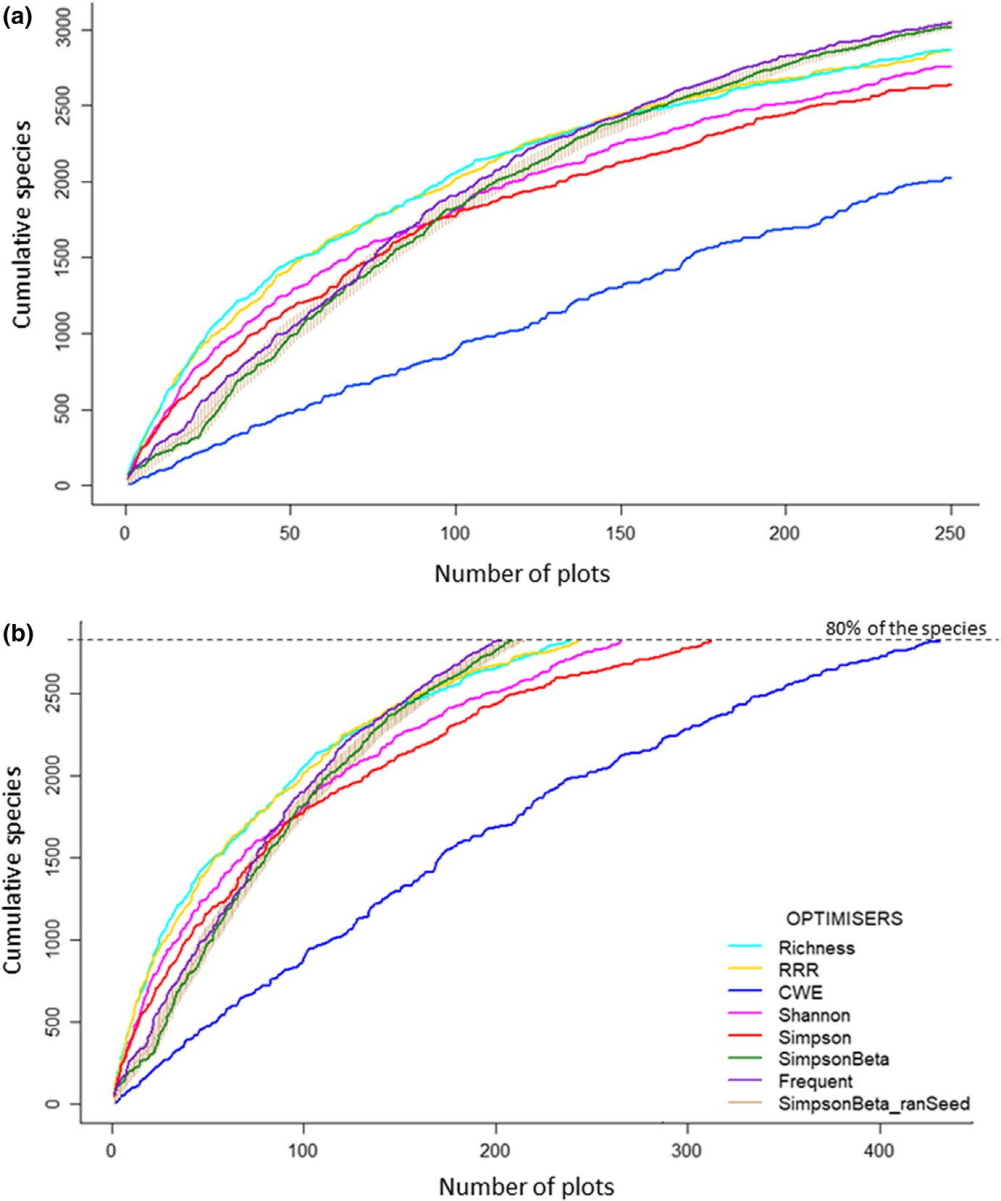
Site optimization process applying conservation reserve design strategies based on: (a) Maximum coverage problem (selection of 250 sites) and (b) Minimum set problem (selection of the minimum number of plots that allow including 80% of the species, represented by dashed line). Optimization has been performed in both cases employing different optimizers, including species richness, range rarity richness (RRR), corrected range rarity richness (CRRR), Shannon‐Wienner diversity index (Shannon), Simpson diversity index (Simpson), the turnover component of beta diversity, or pairwise Simpson dissimilarity index (Simpson beta), the most frequent plots selected in 1,000 iterations with a randomized starting seed using the pairwise Simpson dissimilarity index (frequent), and the plots selected with a randomized seed using the pairwise Simpson dissimilarity index (SimpsonBeta_random seed)

When comparing species accumulated with each of the optimizers, we observed that the species turnover‐based metrics (i.e., the pairwise Simpson dissimilarity with its three implementation variants: Simpson_Beta, Simpson_Random, and Frequent) were the indices that maximized the cumulative number of species (Figure 1). In particular, the Frequent variant outperformed the other two, with 3,051 species accumulated (86.5% of the species recorded; Appendix S6).

For univariate indices, the subsets of plots selected by RRR and species richness when applying the maximal coverage problem, accumulated a greater number of species (2,866 and 2,864, respectively, which accounted for 81.2% of all the species recorded in AusPlots sampling) than the rest of the optimizers. When incorporating species frequencies, the Shannon index outperformed the Simpson index for both the maximal coverage problem and the minimum set problem. CRRR was a poor optimizer, with 2,024 species accumulated which accounted for 57.4% of the total number of species recorded (Figure 1; Appendix S6).

### 3.3 Spatial coverage representativeness

**FIGURE 2:**
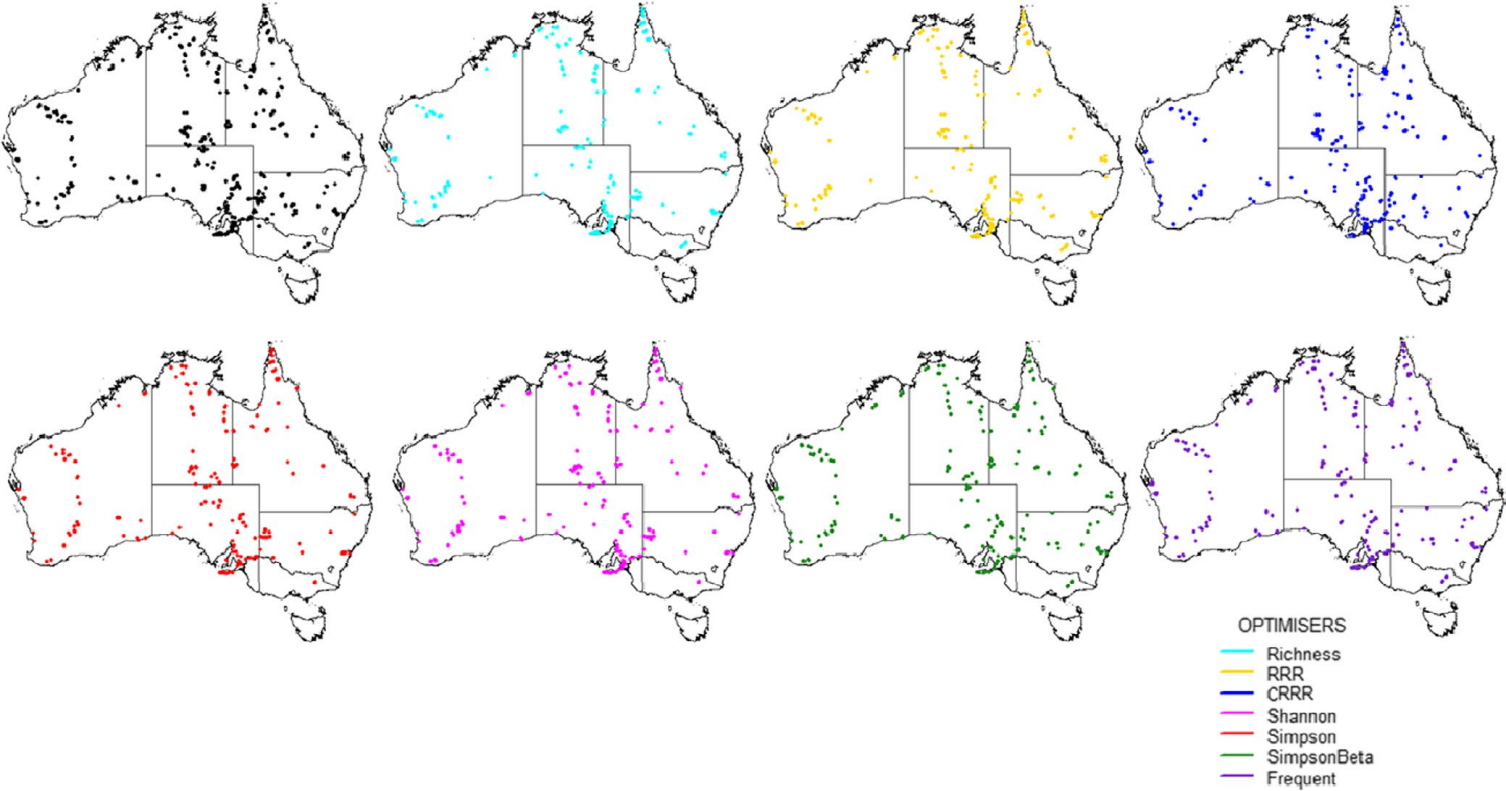
Geographic location of the selected plots (*N* = 250) applying the maximum coverage problem. Black dots correspond to all the plots established. Color dots refer to each of the selection employing different optimizers

All the subsets of plots selected were spatially clustered, but they differed among each other regarding their spatial representativeness. To visually complement the results from the Clark‐Evan test, we mapped the subsets of plots selected with different optimizers (Figure 2). Species richness was the optimizer that presented the most clustered spatial distribution (*R* = 0.366), followed by Shannon and Simpson optimizers (both displaying *R* = 0.408). Plots selected with RRR and CRRR displayed Clark‐Evans values of *R* = 0.414 and *R* = 0.428, respectively. From the species turnover‐based metrics, pairwise Simpson dissimilarity (Simpson_Beta) showed better spatial coverage (*R* = 0.450), whereas the best optimizer in terms of spatial representativeness was Frequent (*R* = 0.545).

Plot selection based on species richness and RRR was geographically biased toward coastal regions, failing to cover remote areas within the Australian outback. This was also the case for the Shannon and Simpson indices. Contrarily, the opposite trend (i.e., optimized plots located toward central and remote areas) was found when selecting plots based on CRRR. This suggests that when selecting plots using univariate diversity metrics, the results are geographically biased toward sites located either in biodiversity hotspots and areas with milder environmental conditions (e.g., richness) or in remote centers of endemism (e.g., CRRR). Plot selection with pairwise Simpson dissimilarity accounts for the species ID and the turnover component; therefore, the spatial distributions displayed with these indices were more balanced in terms of representation across the whole Australia, than those obtained by univariate biodiversity metrics. This trend was enhanced when selecting the most frequent plots after 1,000 simulations of the pairwise Simpson dissimilarity plot selection.

### 3.4 Environmental coverage representativeness

**FIGURE 3:**
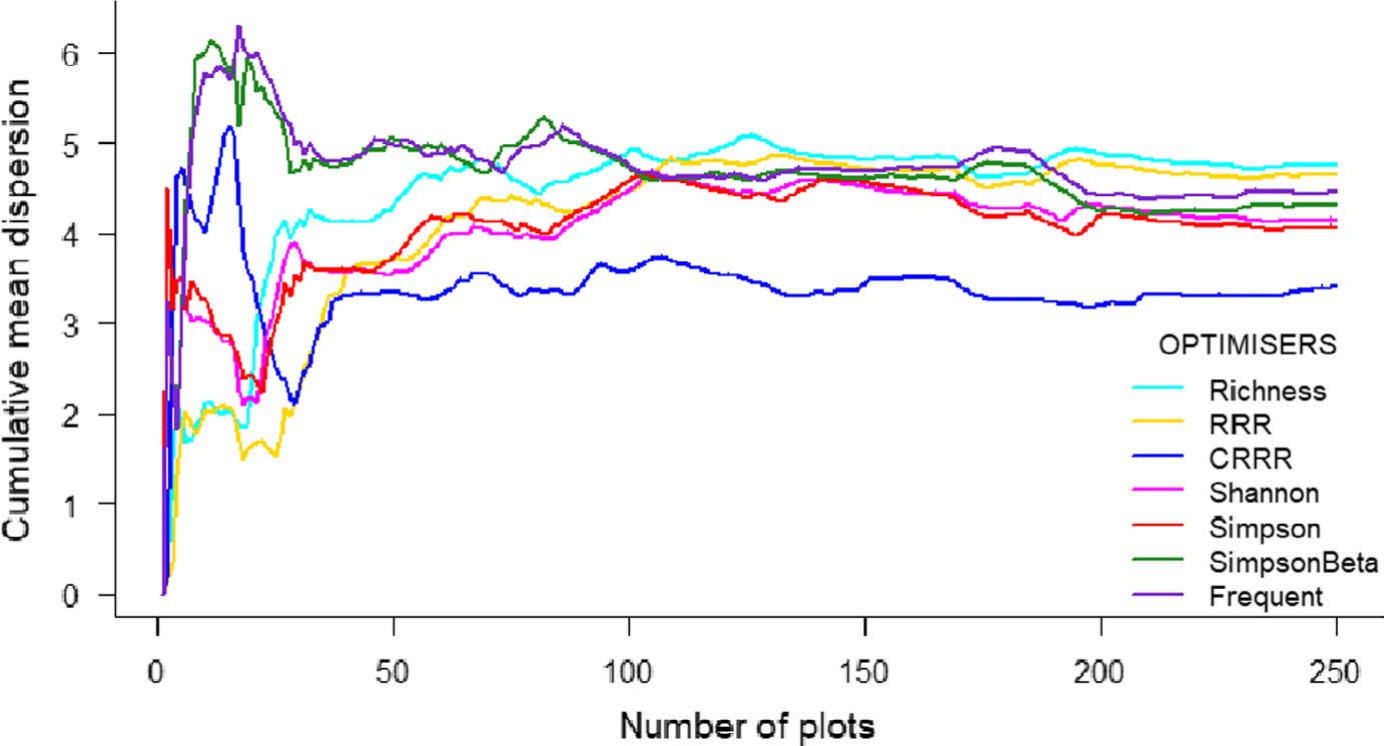
Environmental representativeness of the 250 selected plots using different optimizers reflected by the cumulative mean dispersion. All environmental variables employed in the analyses are described in the Appendix S4

The permutation test for homogeneity of multivariate dispersions showed significant differences in environmental representativeness among optimizers (*F* = 6.49; *p*‐value ≤ .001; Table 2). We found that optimization with CRRR was the least representative in terms of environmental coverage (CRRR: average distance to median = 3.41), showing significant differences with the environmental coverage of the subsets selected by all the other optimizers. Richness and RRR were the most representative with regards to environmental coverage (Richness and RRR: average distance to median = 4.75 and 4.64, respectively), followed by Simpson_Beta and Frequent (Simpson_Beta and Frequent: average distance to median = 4.33 and 4.46, respectively; Figure 3; Table 2), with only marginally significant differences between Richness and Simson_Beta (Table 2). Shannon and Simpson (Shannon and Simpson: average distance to median = 4.15 and 4.07, respectively) were both significantly less environmentally representative than Richness and RRR, while no significant differences were found between the former two and the results obtained by Simpson_Beta and Frequent. Hence, richness was the biodiversity metric that best covered environmental differences when used as the optimizer. Results of the monitoring strategy optimization for plot clusters are detailed in Appendix S7.

**TABLE 2:** Pairwise comparisons between optimizers with regard to environmental representativeness when applying maximal coverage problem at plot level

	Richness	RRR	CRRR	Shannon	Simpson	Simpson_Beta	Frequent
Richness		0.68	**≤0.001**	**≤0.01**	**≤0.05**	≤0.1	0.25
RRR	0.68		**≤0.001**	≤0.1	**≤0.05**	0.20	0.49
CRRR	**≤0.001**	**≤0.001**		**≤0.01**	**≤0.01**	**≤0.001**	**≤0.001**
Shannon	**≤0.05**	≤0.1	**≤0.01**		0.78	0.48	0.21
Simpson	**≤0.01**	**≤0.05**	**≤0.01**	0.75		0.29	0.12
Simpson_Beta	≤0.1	0.22	**≤0.001**	0.48	0.30		0.58
Frequent	0.25	0.48	**≤0.001**	0.21	0.12	0.58	

Note

The observed *p*‐value are located in the below diagonal, while the permuted *p*‐value are in the above diagonal. Only significant differences are highlighted in bold. Notice that marginally significant values (*p*‐value ≤ .1) are shown although not highlighted.

## 4 DISCUSSION

Large‐scale monitoring networks aim for high spatial coverage but resource constraints enforce trade‐offs between spatial and temporal sampling. Our results, as applied to the TERN AusPlots dataset, clearly demonstrate that to design monitoring strategies that track the greatest number of species while ensuring environmental and spatial representativity, it is better to focus on the turnover component (i.e., species replacement) through diversity partitioning than on univariate diversity indices.

Species turnover best optimized the selection of plots from a monitoring network to be revisited more often when applying both the minimum set and the maximal coverage approaches. Species turnover maximized species complementarity and spatial representativeness, without being significantly worse than the other optimizers regarding environmental representativeness. We obtained more robust results when we ran 1,000 random‐seed iterations and extracted the most frequently selected plots (“Frequent”) compared to using a predetermined, fixed seed (“Simpson_Beta”). These results make sense considering that turnover was the most relevant source of change (accounting for 99% of total multisite beta diversity) among the Australian vegetation communities sampled. It is surprising that range‐rarity‐richness performed badly (worse than random) when corrected by species richness (i.e., CRRR), since that should, in theory, highlight complementary sites that have species with few other occurrences.

When comparing univariate biodiversity metrics, our results indicated species richness was the worst performing biodiversity metric optimizer in terms of spatial representativeness. This is consistent with previous findings that have also demonstrated richness‐based decisions, despite their promise and simplicity, do not meet the complementarity principle (Godoy‐Bürki et al., 2014; Gotelli & Colwell, 2001), and are biased toward spatial clustering around more tropical climatic conditions (Veach et al., 2017). Based on these results, we provide further evidence that species richness is not an efficient measure of biodiversity and its change over time (Hillebrand et al., 2018).

Optimization based on corrected range‐rarity‐richness (i.e., CRRR) failed to be environmentally, spatially and ecologically representative in terms of biodiversity, with the lowest number of species accumulated across the whole network (worse than random) and the worst environmental representativeness. The poor performance of optimization based on CRRR has implications for monitoring strategies and conservation planning. While conservation reserves could aim to protect endemic species (Pelletier et al., 2018), monitoring priorities defined on species occurrences at plot level should not be based on CRRR, as it will neither meet the principle of complementarity nor representativeness of the whole network and will fail to inform on the ecological reality.

Among the univariate biodiversity metrics, RRR was the most balanced, capturing a great number of species and being spatially and environmentally representative. Its estimation is straightforward from incidence datasets; therefore, when seeking a simple but relatively reliable way to select sites for a monitoring program, from the univariate metrics we recommend using RRR as an alternative to species turnover‐based prioritization. Our findings are supported by previous work demonstrating the great ecological representation of this index, as well as its effectiveness as a surrogate for biodiversity when fitted to environmental models to predict biodiversity in the absence of available data (Albuquerque & Beier, 2015, 2016).

The Shannon and Simpson (alpha) optimizers performed comparatively poorly in the three dimensions studied here (i.e., ecological, spatial and environmental representativeness). Hence, plot selection prioritization processes should preferably not be based on these metrics.

Both reserve design approaches (minimum set problem and maximal coverage problem) displayed similar results in terms of species accumulation, but with important consideration of the threshold *a priori* selected regardless of the approach. For example, as observed in Figure 1, species accumulations curves for some metrics crossed‐over when reaching approximately either the 70% of the species (minimum set problem) or 150 plots (maximal coverage problem). This suggests the target matters and robust results may be jeopardized if thresholds are set too low.

When implementing optimization for spatial clusters of plots, differences in ecological representativeness were diluted relative to plot‐by‐plot selection (except in the case of CRRR, which still performed significantly worse than the rest of the optimizers). Nevertheless, selection based on species turnover (most specifically employing the Frequent optimizer) performed best, with Frequent and Simpson_Beta approaches the most, and second most, environmentally and spatially representative, respectively. This has implications for hands‐on applications of the current findings, since the prioritization of clusters of plots will need to be carefully supervised to ensure complementarity and representativeness. We therefore suggest that practitioners perform plot‐by‐plot optimization to get the ideal subset and then apply logistic principles to determine clusters of plots in a given geographic area.

Our results have potential application to conservation reserve design, whereby species turnover metrics could be considered to optimize complementarity and representativeness. Various criteria have been followed to design conservation reserves historically, including maximizing species richness or genetic diversity, protecting rare or endemic species or restoring impacted or degraded areas (Kingsland, 2002; Margules et al., 1982). In this sense, Simpson pairwise dissimilarity is potentially useful as it selects a representative subset of the habitats and flora within a region.

Regarding management recommendations, the target set when applying prioritization strategies to large‐scale monitoring networks should consider a minimum threshold of 70% of the total recorded richness when applying the minimum set problem and a subset including at least 20% of the sites when applying the maximal coverage problem.

The application of the findings reported here has some limitations. For example, optimization and therefore reserve design based on species turnover relies on already available ground data and sampled communities and in some cases this information is incomplete or even non‐existent. The optimization process employed in this study (and the tools developed for the analysis) can be implemented in a variety of studies, and can potentially be extended to similar approaches such as site selection based on phylogenetic or functional alpha and beta diversity. Similarly, it could be used to detect change in ecosystem composition over time in the context of a spatial framework; or within a temporal framework to identify sites with the most dissimilar samples among revisits, that is, sites where vegetation is shifting more rapidly over time. These techniques will enable large‐scale monitoring programs to maximize the value of information at a given resourcing level.

In summary, monitoring ecological state, function, and change over time has become essential at national and continental scales. The selection of sites for regular monitoring based on univariate biodiversity metrics (e.g., richness, CRRR) often fails to meet the principles of complementarity and representativeness. We have therefore developed a practical, free, and easy‐to‐use tool that can be used in any species versus sites dataset. The tool uses a set of alpha and beta diversity metrics to optimize species representation in a subset of monitoring sites to maximize species complementarity and spatial and environmental representation. Our results demonstrate that a representative subset of monitoring sites can be selected by finding the most ecologically dissimilar communities. This approach targets differences in composition instead of focusing on univariate metrics such as species richness, while also capturing spatial and environmental diversity. Long‐term monitoring sampling strategies need to be carefully planned and designed. Applying reserve design approaches based on spatial vegetation compositional differences to maximize coverage constitutes a cost‐effective and easily updated strategy to define monitoring priorities that leverages ground data already collected. This will in turn help policy, decision‐making, and conservation practices ensuring them to be based on accurate information that meets the complementarity and representativeness principles.

## AUTHOR CONTRIBUTIONS

**Irene Martín‐Forés:** Conceptualization (equal); data curation (lead); formal analysis (lead); methodology (lead); software (equal); supervision (equal); writing–original draft (lead); writing–review and editing (lead). **Greg R. Guerin:** Conceptualization (equal); formal analysis (supporting); methodology (supporting); software (equal); supervision (lead); writing–review and editing (supporting). **Samantha E. M. Munroe:** Conceptualization (equal); data curation (supporting); formal analysis (supporting); methodology (supporting); software (equal); writing–review and editing (supporting). **Ben Sparrow:** Conceptualization (equal); funding acquisition (lead); project administration (lead); writing–review and editing (supporting).

## Supporting information

Appendix S1‐S8

Supplementary Material

## Data Availability

This paper uses the AusPlots dataset which is publicly available and can be accessed through the ausplotsR package.
