# Supplementary material for: Applying conservation reserve design strategies to define ecosystem monitoring priorities
Source: Ecol Evol. 2021 Nov 11;11(23):17060–70. doi: 10.1002/ece3.8344 (PMC8668797; doi:10.1002/ece3.8344)
Supplement: Supplementary file 1 — Appendix S1‐S8 [file ECE3-11-17060-s001.docx]

**Supplementary material S1.**

**Rcode**

optim_species <- function(speciesVsitesMatrix, n.plt=250, richness=TRUE, RRR=TRUE, CWE=TRUE, shannon=TRUE, simpson=TRUE, simpson_beta=TRUE, start="fixed", plot_name=NULL, frequent=TRUE, random=FALSE, iterations=10, plot=TRUE, verbose=TRUE) {

############################

#check inputs

if(!is.numeric(speciesVsitesMatrix[,1])) {

message("Non-numeric first column... assuming these are site names.")

rownames(speciesVsitesMatrix) <- speciesVsitesMatrix[,1]

speciesVsitesMatrix <- speciesVsitesMatrix[,-1] #remove the first column

}#if the first column of the dataset corresponds to the site names, then apply site names to the rows and remove the column

if(!(start %in% c("fixed", "defined", "random"))) {stop("Argument 'start' must be character and one of: 'fixed', 'defined' or 'random'")}

if(!is.null(plot_name) && (!plot_name %in% rownames(speciesVsitesMatrix))) {stop("Selected plot_name must match a site/row name in the species~sites data.")}

if(n.plt > nrow(speciesVsitesMatrix)) {

message("You are attempting to optimise more sites that exist in the dataset - trimming to
 maximum.")

n.plt <- nrow(speciesVsitesMatrix)

}

if(any(c(random, richness, RRR, CWE, simpson_beta, frequent))) {

speciesVsitesMatrix_binary <- speciesVsitesMatrix

speciesVsitesMatrix_binary[speciesVsitesMatrix_binary > 0] <- 1 #convert abundances to presences

}

########################

#calls

result <- list()

if(richness){

result$Richness <- Richness.opt(speciesVsitesMatrix_binary, n.plt) #

} #end if richness

if(RRR){

result$RRR <- RRR.opt(speciesVsitesMatrix_binary, n.plt) #

} #end if RRR

if(CWE){

result$CWE <- CWE.opt(speciesVsitesMatrix_binary, n.plt) #

} #end if CWE

if(simpson_beta){

result$SimpsonBeta <- simpson_beta.opt(speciesVsitesMatrix_binary, n.plt, start=start, plot_name, verbose) #

} #end if simpson beta

if(shannon) {

result$Shannon <- Shannon.opt(speciesVsitesMatrix, n.plt) #

} #end if shannon

if(simpson) {

result$Simpson <- Simpson.opt(speciesVsitesMatrix, n.plt) #

} #end if simpson

if(frequent) {

hold <- Frequent_simpson_beta.opt(speciesVsitesMatrix_binary, n.plt, iterations, verbose)

result$Frequent <- hold$Freq

result$SimpsonBeta_randSeed <- hold$simspon_rand

} #end if freqent plots

if(random) {

result$Random <- Random.opt(speciesVsitesMatrix_binary, n.plt, iterations, verbose)

} #end if random

##########################

#wrap up:

if(plot) {

try(plot_opt(result))

} #end if plot

return(result)

} #end function

########################

Richness.opt <- function(speciesVsitesMatrix_binary, n.plt) {

Richness <- rowSums(speciesVsitesMatrix_binary) #simple species richness (sum) per plot

RichnessSort <- rev(sort(Richness))[1:n.plt] #inverse of the sort function to get decreasing order

RichnessMCP <- RichnessSort[1:n.plt] #get top n.plt plots based on Richness

RichnessMCPaccum <- specaccum(speciesVsitesMatrix_binary[names(RichnessMCP),], method="collector") #get a species accumulation curve for these selected plots

return(RichnessMCPaccum)

}

########################

RRR.opt <- function(speciesVsitesMatrix_binary, n.plt) {

RRR <- rowSums(speciesVsitesMatrix_binary/colSums(speciesVsitesMatrix_binary)) #presence/absence matrix with presences divided by frequency of that species

RRRSort <- rev(sort(RRR)) #inverse of the sort function to get decreasing order

RRRMCP <- RRRSort[1:n.plt] #get top n.plt plots based on RRR

RRRMCPaccum <- specaccum(speciesVsitesMatrix_binary[names(RRRMCP),], method="collector") #get a species accumulation curve for these selected plots

return(RRRMCPaccum)

}

########################

CWE.opt <- function(speciesVsitesMatrix_binary, n.plt) {

CWE <-rowSums(speciesVsitesMatrix_binary/colSums(speciesVsitesMatrix_binary))/rowSums (speciesVsitesMatrix_binary) #It's RRR divided by richness

CWESort <- rev(sort(CWE)) #inverse of the sort function to get decreasing order

CWEMCP <- CWESort[1:n.plt] #get top n.plt plots based on CWE

CWEMCPaccum <- specaccum(speciesVsitesMatrix_binary[names(CWEMCP),], method="collector") #get a species accumulation curve for these selected plots

return(CWEMCPaccum)

}

########################

Shannon.opt <- function(speciesVsitesMatrix, n.plt) {

Shannon <- diversity(speciesVsitesMatrix, index = "shannon") #Normal Shannon diversity index using vegan package

ShannonSort <- rev(sort(Shannon)) #inverse of the sort function to get decreasing order

ShannonMCP <- ShannonSort[1:n.plt] #get top n.plt plots based on Shannon-Wienner diversity index

ShannonMCPaccum <- specaccum(speciesVsitesMatrix[names(ShannonMCP),], method="collector") #get a species accumulation curve for these selected plots

return(ShannonMCPaccum)

}

########################

Simpson.opt <- function(speciesVsitesMatrix, n.plt) {

Simpson <- diversity(speciesVsitesMatrix, index = "simpson") #Normal Simpson diversity index using vegan package

SimpsonSort <- rev(sort(Simpson)) #inverse of the sort function to get decreasing order

SimpsonMCP <- SimpsonSort[1:n.plt] #get top n.plt plots based on Shannon-Wienner diversity index

SimpsonMCPaccum <- specaccum(speciesVsitesMatrix[names(SimpsonMCP),], method="collector") #get a species accumulation curve for these selected plots

return(SimpsonMCPaccum)

}

########################

simpson_beta.opt <- function(speciesVsitesMatrix_binary, n.plt, start, plot_name, verbose) { #I added plot_name in case the user wants to define a fixed seed which does not correspond to the richest site

original_matrix <- speciesVsitesMatrix_binary

if (start == "fixed"){

start.plot <- rownames(speciesVsitesMatrix_binary)[which.max(rowSums(speciesVsitesMatrix_binary))] #fixed seed: this is the richest plot

} #end if fixed

if (start == "defined") {

start.plot <- plot_name #defined seed: this a specific plot chose by the user

} #end if defined

if (start == "random") {

start.plot <- sample(rownames(speciesVsitesMatrix_binary), 1) #get a random seed plot

}

result <- list()

n <- 1

result[n] <- start.plot

for(i in 1:(n.plt-1)) {

n <- n + 1

simpson <- as.data.frame(as.matrix(betapart::beta.pair(speciesVsitesMatrix_binary)$beta.sim)) #simpson beta diversity between all pairs (excludes species nestedness)

simpson_unlist <- unlist(simpson[start.plot,]) #get the row for the start plot as a vector with names still attached (cf. as.numeric which strips names)

diss_order <- rev(sort.list(unname(simpson_unlist))) #vector of places along the vector to find highest to lowest values - must unname the vector to use sort.list safely

sort.diss <- simpson[start.plot, diss_order] #create single row data frame holding the vector of dissimilarity comparisons to the seed/start.plot in highest to lowest order

equal_plots <- length(which(unlist(sort.diss) == max(unlist(sort.diss))))

if(equal_plots == 1) {

next.plot.name <- names(sort.diss)[1] #select the first plot in the vector, which after sorting is the most dissimilar

}

if(equal_plots > 1) {

next.plot.name <- sample(names(sort.diss[,1:equal_plots])[(!names(sort.diss[,1:equal_plots]) %in% start.plot)],1) #make a random choice of selected plot out of all that have the same dissimilarity score to avoid alphabetical selection

}

#if(next.plot.name == start.plot) {next.plot.name <- names(sort.diss)[2]}

result[n] <- next.plot.name #add it to the list of plots to save

if(verbose) cat(next.plot.name, " ", sort.diss[,1], "\n") #Print out the chosen plot and its dissimilarity score

speciesVsitesMatrix_binary[start.plot,] <- speciesVsitesMatrix_binary[start.plot,] + speciesVsitesMatrix_binary[next.plot.name,] #Merge the seed plot with the latest selected plot to get all occurrences into one virtual plot

speciesVsitesMatrix_binary[start.plot,][speciesVsitesMatrix_binary[start.plot,] > 0] <- 1 #set all values to 1 so it is PA data in case species are shared

speciesVsitesMatrix_binary <- speciesVsitesMatrix_binary[(!rownames(speciesVsitesMatrix_binary) %in% next.plot.name),]

}

dissimilarplots <- unlist(result) #vector of plot names in order selected

return(specaccum(original_matrix[dissimilarplots,], method="collector"))

}

########################

Frequent_simpson_beta.opt <- function(speciesVsitesMatrix_binary, n.plt, iterations, verbose) {

opt.runs.freq <- list()

n <- 0

for(i in 1:iterations) {

n <- n + 1

if(verbose) cat("Rep ", n, "\n")

opt.runs.freq[[n]] <- simpson_beta.opt(speciesVsitesMatrix_binary, n.plt, start = "random", verbose=verbose)

} #end iterations

freq_plots <- plyr::count(unlist(lapply(opt.runs.freq, FUN=function(x) as.character(names(x$richness)))))

freq_plots <- freq_plots[rev(order(freq_plots$freq)),]

freq_plots <- freq_plots[1:n.plt,]

freq_accum <- specaccum(speciesVsitesMatrix_binary[as.character(freq_plots$x),], method="collector")

#create mean/sd accumulation for simpson iterations with random seed

combined.rand_specaccum <- opt.runs.freq[[1]] #copy one specaccum object in the list of random starts for format

combined_matrix_rand <- do.call(rbind, lapply(opt.runs.freq, function(x) {return(x$richness)})) #compile the cumulative richness results from reps above into a matrix

combined.rand_specaccum$richness <- apply(combined_matrix_rand, 2, mean) #using the matrix, calculate the mean for each additional plot, and add that to the richness slot in the specaccum object

combined.rand_specaccum$sd <- apply(combined_matrix_rand, 2, sd) #same for standard deviation

combined.rand_specaccum$method <- "random" #assign it as random not collector so it plots correctly as mean and SD

freq_accum_lst <- list()

freq_accum_lst$simspon_rand <- combined.rand_specaccum

freq_accum_lst$Freq <- freq_accum

return(freq_accum_lst)

}

####################################

Random.opt <- function(speciesVsitesMatrix_binary, n.plt, iterations, verbose) {

Sppaccum_freq <- list() #

n <- 0

for(i in 1:iterations) {

n <- n + 1

if(verbose) cat("Rep ", n, "\n")

Sppaccum_freq[[n]]<- specaccum(speciesVsitesMatrix_binary[sample(nrow(speciesVsitesMatrix_binary), n.plt),], method="collector")

}

combined.rand_specaccum <- Sppaccum_freq[[1]] #copy one for format

combined_matrix_rand <- do.call(rbind, lapply(Sppaccum_freq, function(x) {return(x$richness)})) #compile the cumulative richness results from reps above into a matrix

combined.rand_specaccum$richness <- apply(combined_matrix_rand, 2, mean) #using the matrix, calculate the mean for each additional plot, and add that to the richness slot in the specaccum object

combined.rand_specaccum$sd <- apply(combined_matrix_rand, 2, sd) #same for standard deviation

combined.rand_specaccum$method <- "random" #assign it as random not collector so it plots correctly as mean and SD

return(combined.rand_specaccum) #object that can be plotted with mean line with bars for sd

}

#################################

plot_opt <- function(optim_result, choices=c("Richness", "RRR", "CWE", "Shannon", "Simpson", "SimpsonBeta", "Frequent", "SimpsonBeta_randSeed", "Random")) {

optim_result <- optim_result[names(optim_result) %in% choices]

plot(1, ylim=c(0, max(unlist(lapply(optim_result, FUN=function(x) max(x$richness))))), xlim=c(0, length(optim_result[[1]]$richness)), type="n", xlab = "Number of plots", ylab = "Cumulative species", main="Site optimisation Maximum Coverage Problem", las=1, bty="l", cex.main=1.2) #blank template plot

opt.col <- sample(rainbow(8), length(which(names(optim_result) %in% choices[1:8]))) #random colours for the plot lines of optimisers, but exclude random for now

zzz <- 0

for(j in optim_result) { #for each optimiser result in the list

zzz <- zzz + 1

if(names(optim_result)[zzz] != "Random") {

plot(optim_result[[zzz]], col=opt.col[zzz], lwd=1.5, add=TRUE) #add line for each optimiser

} #end if not random

if(names(optim_result)[zzz] == "Random") {

plot(optim_result[[zzz]], col="gray", lwd=1, lty=2, add=TRUE) #add line for random

} #end if Random

} #close for j in ...

if("Random" %in% names(optim_result)) {

legend("bottomright", legend=names(optim_result), lty=c(rep(1, (length(optim_result)-1)), 2), col=c(opt.col[1:(length(optim_result)-1)], "gray"), cex=1, bty='n', lwd=rep(3, length(optim_result)))

} #end if Random

if(!"Random" %in% names(optim_result)) {

legend("bottomright", legend=names(optim_result), lty=rep(1, length(optim_result)), col=opt.col[1:length(optim_result)], cex=1, bty='n', lwd=rep(3, length(optim_result)))

} #end if not Random

} #end plot_opt function

**Supplementary material S2. Additional explanation of biodiversity metrics**

Species richness is simply the count of the number of species present in a given site and it is best used as an optimiser when the goal is to identify areas containing the greatest number of unique species. RRR is a rarity-weighted richness calculated as the sum of the inverse of the number of sites in which a species occurs and it is used to identify areas of high biodiversity and biological uniqueness. CWE is calculated as RRR corrected by the species richness and is used to identify centres of endemism highlighting range-restricted species. Shannon combines species richness and evenness or equitability by computing the species’ relative abundances; it assumes that all species are represented in a sample and that they are randomly sampled. Simpson is the complement of Simpson's original dominance index, and represents the probability that two randomly chosen individuals belong to different species. The species turnover-based metric is based on diversity partitioning, which separates species replacement (i.e. turnover) from species loss (i.e. nestedness); it corresponds to the turnover component of the Sorensen dissimilarity and describes spatial turnover without the influence of richness gradients (because unshared species in the larger species sample are disregarded; Lennon et al., 2001; Baselga, 2010). For the species turnover-based metric, as pairwise Simpson dissimilarity differs depending on the plot that is chosen as seed, we computed three variants: fixed seed (Simpson_Beta), in which we selected the plot with the greatest richness as the starting point; random seed (Simpson_Random), in which we randomly selected the plot at the starting point and we iterated this process 1,000 times; and the most frequent plots (Frequent), by computing the most frequently selected pots in the former 1,000 simulations. When employing the pairwise Simpson dissimilarity, the most dissimilar plot to the one used as seed in terms of pairwise Simpson dissimilarity was added by the function. Subsequently the species present in both plots are pooled together and the most dissimilar one to the combination of both is added. This process was repeated until reaching the top 250 plots that maximised species turnover.

**Supplementary material S3.** Example of the comparison of different optimisers after applying the optim_species function to an alternative dataset. In this case the dataset corresponds to plots located in South Australia within the frame of the project Transect for Environmental Monitoring and Decision Making (TREND). We applied the maximal coverage problem selecting 85 plots to optimise.


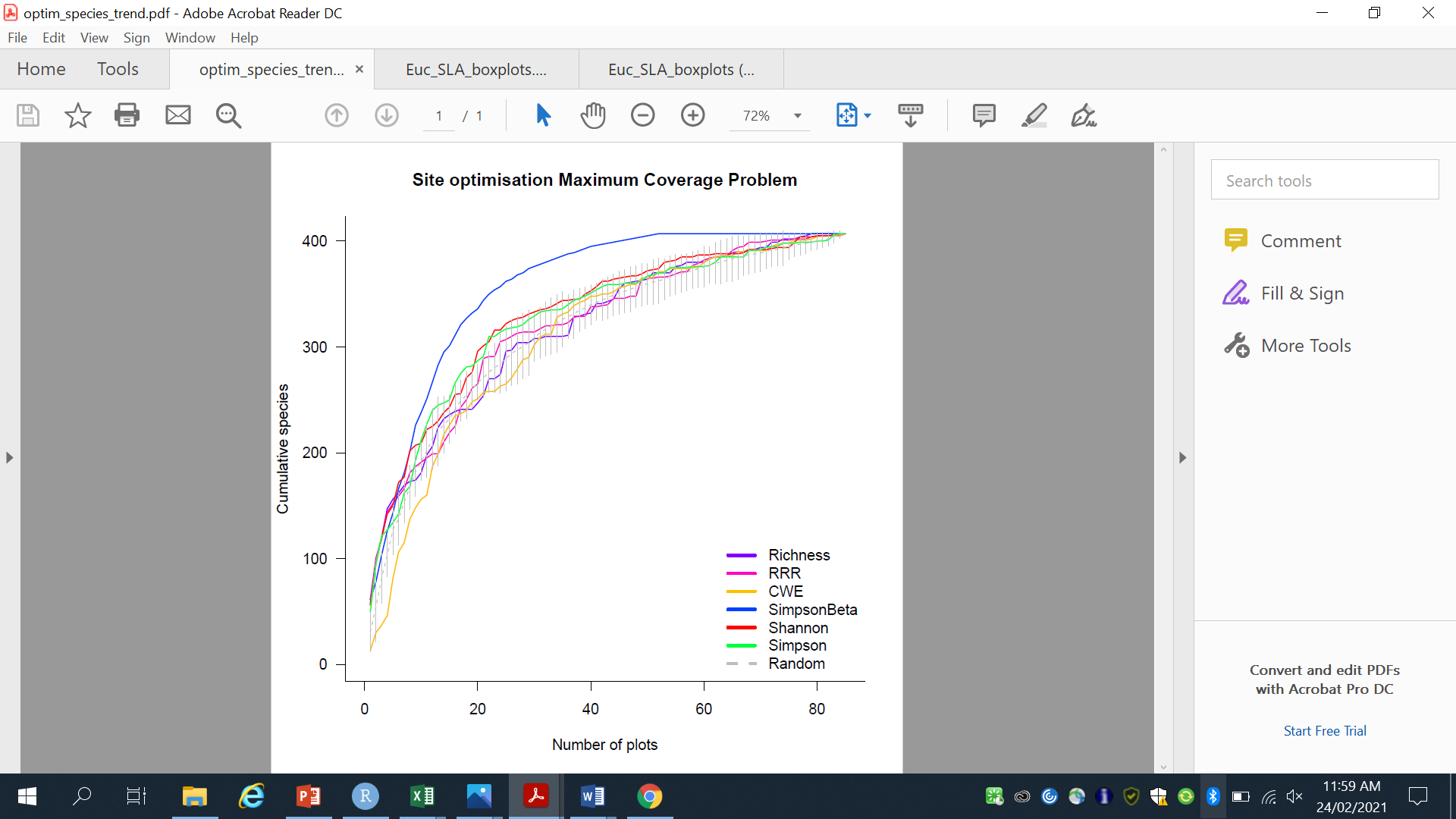


**Supplementary material S4. Climate variables employed to calculate the environmental representativeness of the selected plots. The variables were obtained from Harwood et al. (2016)**

| **Code** | **Brief description** |
| --- | --- |
| *Aridity index* | |
| ADI | Minimum monthly aridity index (proportion) |
| ADM | Mean annual aridity index (annual precipitation/annual potential evaporation) (proportion) |
| ADX | Maximum monthly aridity index (proportion) |
| *Evaporation* | |
| EPA | Annual potential evaporation (mm) |
| EPI | Minimum monthly potential evaporation (mm) |
| EPX | Maximum monthly potential evaporation (mm) |
| EAA | Annual total actual evapotranspiration terrain scaled using MODIS (mm) |
| EAAS | Annual total actual evapotranspiration modelled using terrain-scaled water holding capacity (mm) |
| *Maximum temperature* | |
| TXM | Maximum temperature – Annual mean °C |
| TXI | Maximum temperature - monthly minimum °C |
| TXX | Maximum temperature - monthly maximum °C |
| *Minimum temperature* | |
| TNM | Minimum temperature – Annual mean °C |
| TNI | Minimum temperature - monthly minimum °C |
| TNX | Minimum temperature - monthly maximum °C |
| *Temperature range* | |
| TRI | Minimum monthly mean diurnal temperature range °C |
| TRX | Maximum monthly mean diurnal temperature range °C |
| TRA | Annual temperature range (TXX – TNI) °C |
| *Precipitation* | |
| PTA | Annual precipitation mm |
| PTI | Minimum monthly precipitation mm |
| PTX | Maximum monthly precipitation mm |
| PTS1 | Precipitation seasonality 1- solstice seasonality composite factor ratio |
| PTS2 | Precipitation seasonality 2- equinox seasonality composite factor ratio |
| *Water deficit* | |
| WDA | Annual atmospheric water deficit (annual precipitation – annual potential evaporation) mm |
| WDI | Minimum monthly atmospheric water deficit (precipitation - potential evaporation) mm |
| WDX | Maximum monthly atmospheric water deficit (precipitation - potential evaporation) mm |

**Supplementary material S5.**
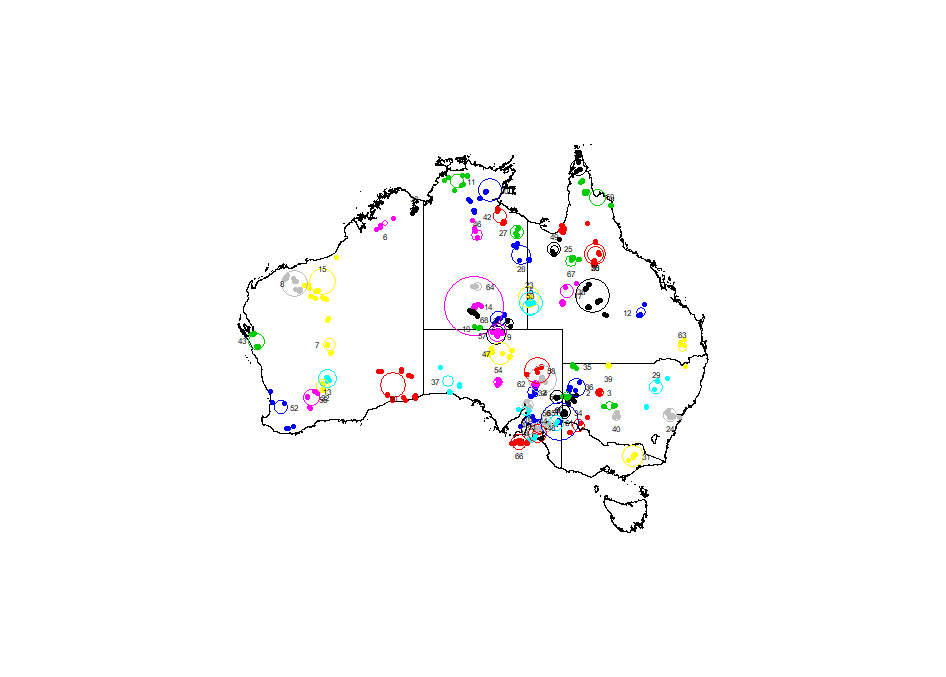


**S5-Fig. 1.** Map representing geographic clusters of plots to translate the findings into a realistic and feasible approach. Colours indicate different clusters, number indicate the cluster ID (see Supplementary material S3) and the size of the circle indicates the number of plots within each cluster.

**S5-Table 1. Clusters of plots, coordinates of their centroids and number of plots within them**

| Cluster ID | Number of plots | Number of sites | Latitude | Longitude |
| --- | --- | --- | --- | --- |
| clu1 | 12 | 12 | -11.9637 | 142.3816 |
| clu2 | 6 | 6 | -31.4776 | 144.2586 |
| clu3 | 6 | 6 | -32.6306 | 145.1296 |
| clu4 | 8 | 5 | -31.4964 | 138.544 |
| clu5 | 17 | 6 | -23.7562 | 138.3792 |
| clu6 | 4 | 4 | -16.8637 | 125.6894 |
| clu7 | 9 | 8 | -27.353 | 120.8142 |
| clu8 | 19 | 19 | -22.0562 | 117.8034 |
| clu9 | 7 | 7 | -25.5573 | 136.4409 |
| clu10 | 18 | 18 | -30.8827 | 126.355 |
| clu11 | 10 | 10 | -13.1988 | 131.9348 |
| clu12 | 7 | 7 | -24.568 | 147.8653 |
| clu13 | 13 | 13 | -30.3101 | 120.6808 |
| clu14 | 42 | 18 | -24.0693 | 133.4427 |
| clu15 | 19 | 19 | -21.8701 | 120.2695 |
| clu16 | 8 | 4 | -32.6554 | 138.0497 |
| clu17 | 24 | 24 | -23.1361 | 143.6343 |
| clu18 | 13 | 7 | -35.0169 | 138.8766 |
| clu19 | 4 | 4 | -25.9328 | 133.86 |
| clu20 | 17 | 17 | -13.9922 | 134.7901 |
| clu21 | 7 | 4 | -35.5107 | 138.4406 |
| clu22 | 12 | 12 | -31.9722 | 119.3232 |
| clu23 | 15 | 5 | -23.2451 | 138.2123 |
| clu24 | 10 | 9 | -33.4671 | 150.4113 |
| clu25 | 6 | 6 | -19.079 | 140.3453 |
| clu26 | 12 | 12 | -19.5532 | 143.9305 |
| clu27 | 10 | 10 | -17.6146 | 137.0923 |
| clu28 | 14 | 14 | -19.6014 | 137.4888 |
| clu29 | 10 | 10 | -31.0635 | 149.1966 |
| clu30 | 13 | 13 | -26.3325 | 135.3958 |
| clu31 | 15 | 15 | -37.0249 | 147.163 |
| clu32 | 20 | 10 | -30.4005 | 139.3001 |
| clu33 | 4 | 4 | -15.7952 | 128.1739 |
| clu34 | 9 | 9 | -34.3382 | 142.4097 |
| clu35 | 4 | 4 | -29.2578 | 142.0513 |
| clu36 | 13 | 11 | -31.0322 | 142.2075 |
| clu37 | 8 | 8 | -30.5226 | 131.1769 |
| clu38 | 10 | 10 | -22.7827 | 141.4962 |
| clu39 | 4 | 4 | -29.2031 | 145.0439 |
| clu40 | 6 | 6 | -33.5424 | 145.6879 |
| clu41 | 10 | 6 | -31.9441 | 140.5602 |
| clu42 | 10 | 10 | -16.27 | 135.6802 |
| clu43 | 12 | 9 | -27.0428 | 114.4813 |
| clu44 | 7 | 4 | -33.951 | 138.207 |
| clu45 | 7 | 6 | -33.1889 | 137.8196 |
| clu46 | 8 | 8 | -17.9138 | 133.6234 |
| clu47 | 16 | 16 | -28.1194 | 135.6589 |
| clu48 | 8 | 4 | -34.5859 | 138.8827 |
| clu49 | 10 | 10 | -19.079 | 140.3453 |
| clu50 | 15 | 15 | -19.5532 | 143.9305 |
| clu51 | 7 | 7 | -31.8375 | 141.4666 |
| clu52 | 10 | 10 | -32.7933 | 116.6572 |
| clu53 | 12 | 4 | -24.1515 | 138.2374 |
| clu54 | 7 | 7 | -30.5942 | 135.4801 |
| clu55 | 10 | 10 | -30.9752 | 120.2899 |
| clu56 | 11 | 8 | -34.2875 | 139.6342 |
| clu57 | 14 | 14 | -26.5941 | 135.2836 |
| clu58 | 19 | 11 | -29.5789 | 138.9052 |
| clu59 | 12 | 12 | -14.5616 | 144.045 |
| clu60 | 27 | 16 | -34.0285 | 140.7325 |
| clu61 | 11 | 7 | -34.1447 | 140.4778 |
| clu62 | 6 | 3 | -30.7738 | 138.67 |
| clu63 | 6 | 6 | -27.5596 | 151.4464 |
| clu64 | 6 | 6 | -22.29 | 133.654 |
| clu65 | 10 | 10 | -33.2362 | 141.2395 |
| clu66 | 11 | 11 | -35.8077 | 137.3207 |
| clu67 | 8 | 8 | -20.1054 | 141.8365 |
| clu68 | 11 | 11 | -25.1547 | 135.4541 |

**Supplementary material S6.** Species accumulated when applying the maximum coverage problem to select a subset of 250 plots to revisit. For details of selected plots see associated datasets in Dryad (Martin-Fores et al. 2021).

| **Optimiser** | **Number of plots** | **Number of sites** | **Species accumulated** | **% of total species** |
| --- | --- | --- | --- | --- |
| Richness | 250 | 229 | 2864 | 81.2% |
| RRR | 250 | 233 | 2866 | 81.2% |
| CWE | 250 | 235 | 2024 | 57.4% |
| Shannon | 250 | 231 | 2756 | 78.1% |
| Simpson | 250 | 234 | 2633 | 74.6% |
| Simpson_Beta | 250 | 245 | 3021 | 85.6% |
| Frequent | 250 | 245 | 3051 | 86.5% |
| Simpson_Random | 250 |  | 3030.9 ± 14.7 | 85.9% |

**Supplementary material S7. Results of monitoring strategy optimisation for plot clusters**

The results obtained when analysing clusters of plots differed from those obtained for single plots. In terms of ecological representativeness, the differences in species accumulation among most of the different optimisers become diluted when selecting clusters of plots instead of single plots. The only biodiversity metric that had a considerably worse performance was CWE (S7-Fig. 1).

Regarding spatial representativeness, when selecting clusters of plots, we obtained opposite trends than when selecting single plots. Species richness was the best optimiser regarding spatial representativeness (R = 0.218), followed by RRR, Frequent and CWE optimisers (R = 0.188, R = 0.184 and R = 0.172). Pairwise Simpson dissimilarity (Simpson_Beta) showed Clark-Evans values of R = 0.134. Clusters of plots selected with Shannon and Simpson metrics displayed both the most clustered spatial coverages (R = 0.011 and R = 0.009, respectively; S7-Fig. 2; Supplementary material S8).

The most environmentally representative subset of selected clusters was obtained using species turnover, with Frequent the best optimiser in terms of environmental representation (Frequent: average distance to median = 4.753, respectively; S7-Fig. 1; S7-Table 1), followed by RRR, Simpson_Beta and Simpson (RRR, Simpson_Beta and Simpson: average distance to median = 4.56, 4.55 and 4.53, respectively), with no significant differences among them. ‘Frequent’ displayed marginally significantly better environmental representativeness than Shannon (Shannon: 4.34), whereas all the four former biodiversity metrics performed significantly (for Frequent) or marginally significantly (for the later three) better than richness in terms of environmental representativeness (Richness: 4.14). Finally, all the optimisers performed significantly better than CWE in terms of environmental representativeness (CWE: average distance to median = 3.60; S7-Fig. 1; S7-Table 1).


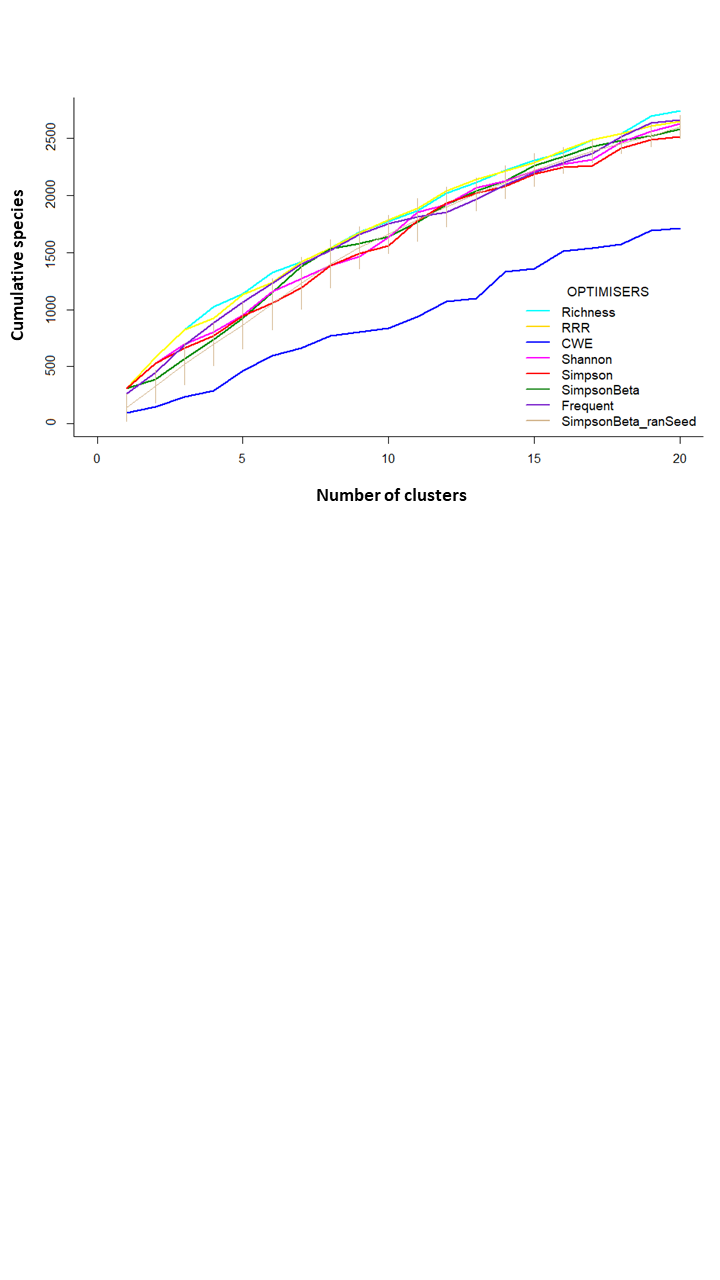

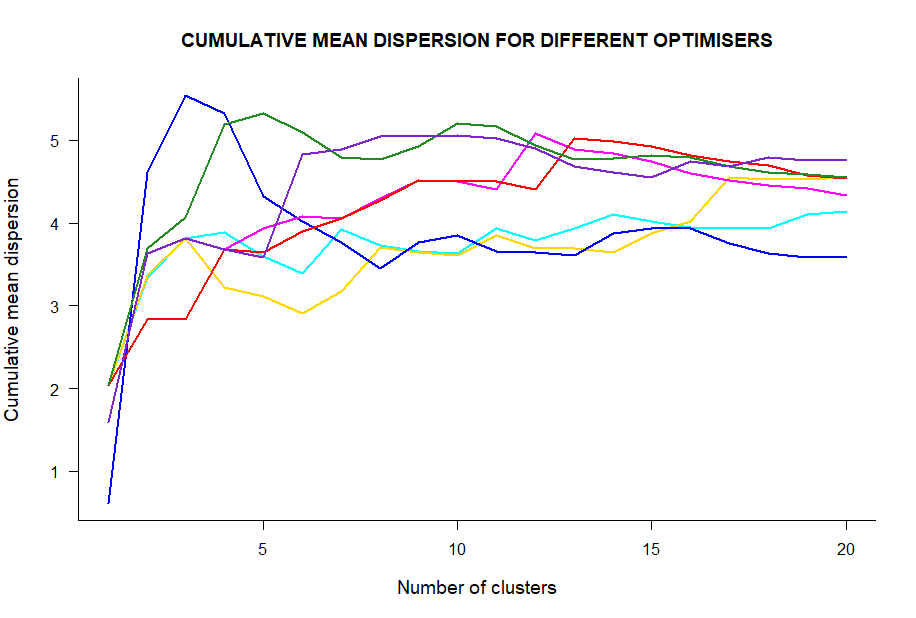
 **S7-Fig. 1.** a) Site optimisation process applying conservation reserve design strategies based on Maximum coverage problem (selection of 20 clusters); b) environmental representativeness of the 20 selected clusters using different optimisers reflected by the cumulative mean dispersion

**a)**

**b)**


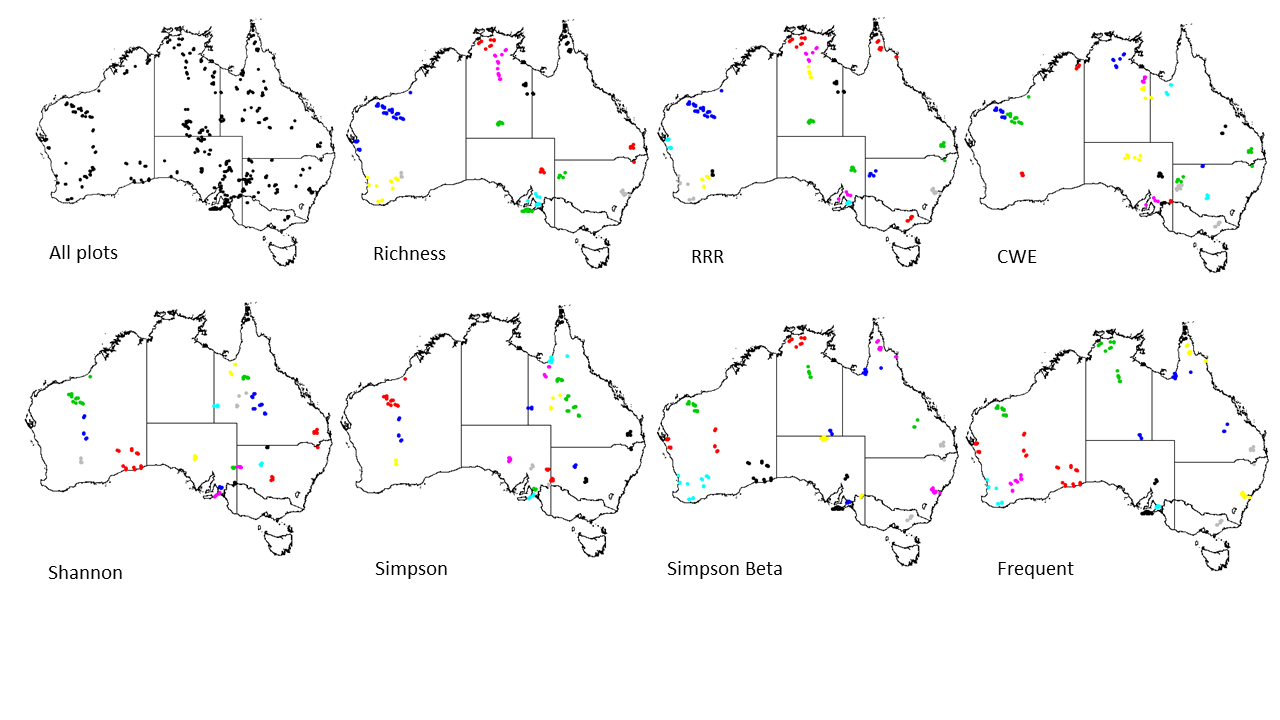


**S7-Fig. 2.** Geographic location of the selected clusters (n = 20) applying the maximal coverage problem. Black dots correspond to all the plots established. Colour dots refer to each of the selection employing different optimisers.

**S7-Table 1. Pairwise comparisons between optimisers with regards to environmental representativeness when applying maximal coverage problem selecting spatial clusters. The observed p-value are located in the below diagonal, while the permuted p-value are in the above diagonal.** Only significant differences are highlighted in bold. Notice that marginally significant values (p-value ≤0.1) are shown although not highlighted.

|  | Richness | RRR | CWE | Shannon | Simpson | SimpsonBeta | Frequent |
| --- | --- | --- | --- | --- | --- | --- | --- |
| Richness |  | ≤0.1 | **≤0.05** | **≤0.001** | **≤0.001** | **≤0.05** | **≤0.01** |
| RRR | ≤0.1 |  | **≤0.01** | **≤0.001** | **≤0.001** | 0.99 | 0.42 |
| CWE | **≤0.05** | **≤0.001** |  | **≤0.001** | **≤0.05** | **≤0.001** | **≤0.001** |
| Shannon | **≤0.001** | **≤0.001** | **≤0.01** |  | 0.36 | **≤0.001** | **≤0.001** |
| Simpson | **≤0.001** | **≤0.001** | **≤0.05** | 0.35 |  | **≤0.001** | **≤0.001** |
| SimpsonBeta | **≤0.05** | 0.99 | **≤0.001** | **≤0.001** | **≤0.001** |  | 0.39 |
| Frequent | **≤0.05** | 0.42 | **≤0.001** | **≤0.001** | **≤0.001** | 0.41 |  |

**Supplementary material S8.** Selected clusters with the different optimisers, number of sites and plots included and species accumulated

| **Optimiser** | **Clusters selected** | **Number of plots** | **Number of sites** | **Species accumulation** |
| --- | --- | --- | --- | --- |
| Richness | clu59, clu52, clu8, clu18, clu15, clu43, clu1, clu14, clu55, clu22, clu24,clu36, clu20, clu11, clu28, clu46, clu63, clu32, clu66, clu44 | 278 | 230 | 2645 |
| RRR | clu59, clu8, clu52, clu15, clu43, clu14, clu18, clu1, clu55, clu36, clu20, clu28, clu63, clu22, clu24, clu11, clu31, clu32, clu46, clu44 | 282 | 234 | 2693 |
| CWE | clu56, clu31, clu63, clu39, clu60, clu12, clu40, clu36, clu8, clu28, clu49, clu27, clu25, clu20, clu33, clu44, clu47, clu13, clu32, clu15 | 245 | 216 | 1698 |
| Shannon | clu59, clu46, clu1, clu66, clu63, clu44, clu11, clu24, clu55, clu43, clu36, clu31, clu51, clu37, clu52, clu18, clu47, clu48, clu22, clu49 | 210 | 173 | 2435 |
| Simpson | clu59, clu46, clu1, clu66, clu11, clu63, clu44, clu55, clu24, clu43, clu37, clu47, clu31, clu25, clu48, clu12, clu9, clu51, clu36, clu52 | 195 | 184 | 2351 |
| Simpson_Beta | clu59, clu61, clu66, clu31, clu22, clu30, clu52, clu24, clu8, clu11, clu63, clu43, clu4, clu9, clu50, clu18, clu46, clu10, clu12, clu7 | 227 | 212 | 2628 |
| Frequent | clu8, clu66, clu52, clu50, clu43, clu31, clu24, clu11, clu46, clu18, clu63, clu7, clu12, clu10, clu55, clu1, clu9, clu59, clu22, clu4 | 225 | 213 | 2706 |
| Simpson_Random |  |  |  | 2626.5 ± 49.8 |
