# Supplementary material for: Applying conservation reserve design strategies to define ecosystem monitoring priorities
Source: Ecol Evol. 2021 Nov 11;11(23):17060–70. doi: 10.1002/ece3.8344 (PMC8668797; doi:10.1002/ece3.8344)
Supplement: Supplementary file 2 — Supplementary Material [file ECE3-11-17060-s002.pdf]

Package ‘ausplotsR’

March 17, 2021

Type Package

Title TERN AusPlots Analysis Package

Version 1.2.3

Date 2021-3-12

Maintainer Greg Guerin <ggueri01@gmail.com>

Depends R (>= 3.1.0), vegan, maps, mapdata

Suggests knitr, rmarkdown

Imports plyr, R.utils, simba, httr, jsonlite, sp, maptools, ggplot2,
gtools, jose, betapart, curl

Description Extraction, preparation, visualisation and analysis of TERN AusPlots ecosystem monitoring data. Direct access to plot-based data on vegetation and soils across Australia, including physical sample barcode numbers. Simple function calls extract the data and merge them into species occurrence matrices for downstream analysis, or calculate things like basal area and fractional cover. TERN AusPlots is a national field plot-based ecosystem surveillance monitoring method and dataset for Australia. The data have been collected across a national network of plots and transects by the Terrestrial Ecosystem Research Network (TERN - <https://www.tern.org.au>), an Australian Government NCRIS-enabled project, and its Ecosystem Surveillance platform (<https://www.tern.org.au/tern-observatory/tern-ecosystem-surveillance/>).

License GPL-3

LazyData TRUE

VignetteBuilder knitr

RoxygenNote 7.1.1

Encoding UTF-8

NeedsCompilation no

Author Greg Guerin [aut, cre],
Tom Saleeba [aut],
Samantha Munroe [aut],
Bernardo Blanco-Martin [aut],
Irene Martín-Forés [aut],
Andrew Tokmakoff [aut]

Repository CRAN

Date/Publication 2021-03-17 09:30:09 UTC

R topics documented:

ausplotsR-package 2
ausplots_visual 5
basal_area 7
fractional_cover 8
get_ausplots 10
growth_form_table 13
optim_species 16
plot_opt 19
single_cover_value 21
species_list 23
species_table 24

Index **27**

`ausplotsR-package` *Live extraction, preparation, visualisation and analysis of TERN AusPlots ecosystem monitoring data.*

Description

Through ausplotsR, users can directly access plot-based data on vegetation and soils across Australia, including physical sample barcode numbers. Simple function calls extract the data and merge them into species occurrence matrices for downstream analysis or calculate things like basal area and fractional cover.

Details

Package:	ausplotsR
Type:	Package
Version:	1.2.3
Date:	2021-3-12
License:	GPL-3

TERN AusPlots is a national field plot-based ecosystem surveillance monitoring method and dataset for Australia. A standardised national network of plots enables consistent ecological assessment and monitoring. The data have been collected across a national network of plots and transects by the Terrestrial Ecosystem Research Network (TERN - https://www.tern.org.au), an Australian Government NCRIS-enabled project, and its Ecosystem Surveillance platform (https://www.tern.org.au/tern-observatory/tern-ecosystem-surveillance/).

The AusPlots survey method is made up of a series of individual modules, which are described

in full in the protocols manual (White et al. 2012, Sparrow et al. 2020). Data from the following modules can currently be accessed through ausplotsR: plot selection and layout; vascular plant vouchering; point intercept; basal area; structural summary; and soils. Field data are recorded directly onto mobile (tablet) devices before being stored in cloud-based server infrastructure.

Method summaries:

Plot layout: 1 ha (100 x 100 m) plots are permanently marked over a homogenous patch of terrestrial vegetation.

Soil sampling: soils are characterised in a pit at the SW corner of the plot to 1 m depth and 500 g samples are collected at 10 cm depth intervals; soil bulk density is measured; nine soil subsites are located across the plots and 500 g samples are taken at 10 cm depths to 30 cm. Additionally, 200 g surface soil (~3 cm depth) samples are taken at the nine subsites and silica-dried for metagenomic analysis.

Structural summary: the dominant species in three vegetation strata (lower, middle and upper) are identified visually by the observer. The vegetation is then categorised into MVGs according to the Australian National Vegetation Information System (NVIS 2017).

Vascular plant vouchering: all vascular plants within the plot are detected visually, with a herbarium voucher taken for each unique taxon, which is assigned a barcode and identified then permanently stored in a herbarium. Additionally, silica-dried material is sampled from each species for genetic and isotopic analysis, and five replicate samples are taken for dominant species. Bryophytes are sometimes recorded opportunistically (e.g. when prominent) but full diversity is not systematically collected.

Point-intercept : 10 x 100 m long transects are laid out within the plot in a grid pattern. A staff with laser pointer and densiometer is used to record species, growth form and height plus substrate type every 1 m along the transects, resulting in a total of 1010 (10 x 101) point-intercept hits for the plot.

Basal wedge : a basal wedge is used to determine the basal area of trees and shrubs at 1.3 m, with measures of hits per species taken at nine locations across the plot.

Additional modules in the AusPlots method not available through this package include: full DGPS coordinate data (only the coordinates of the SW corner are provided here), photo panoramas, Leaf Area Index.

Acknowledging TERN AusPlots data in publications:

It is important that users of data extracted via ausplotsR acknowledge TERN, the AusPlots dataset and ausplotsR by including an appropriate citation in the Acknowledgments or References section of any published papers/reports/theses that make use of the data. To make this easy, a data citation is automatically generated when you extract data (see `get_ausplots`). It has the following format:

TERN ("year") AusPlots ecosystem surveillance monitoring dataset (URL: http://aekos.org.au/). Obtained via the ausplotsR R package (URL: https://github.com/ternaustralia/ausplotsR), accessed "day month year".

To cite the ausplotsR package itself and the functions it contains, enter: `citation('ausplotsR')`, or see links to citable archived versions with DOI at https://github.com/ternaustralia/ausplotsR.

Key functions in the ausplotsR package:

`get_ausplots` : This function is the starting point for accessing data through the ausplotsR package for the following survey modules: Site information; Vegetation point intercept; Vegetation vouchers; Basal wedge; Vegetation structural summaries; Soils and soil samples information.

`ausplots_visual` : Quick maps and demonstration graphics for AusPlots.

`species_table` : Species occurrence matrices for AusPlots.

`growth_form_table` : Plant growth form occurrence matrices for TERN AusPlots.

`fractional_cover` : Fractional cover based on TERN AusPlots data.

`basal_area` : Basal area of tree trunks in TERN AusPlots.

`single_cover_value` : Vegetation cover in TERN AusPlots by height and growth form subsets.

`species_list` : Simplified plant species lists for TERN AusPlots based on verified vouchers.

`optim_species` : Species accumulation optimisation for community data including TERN AusPlots.

Author(s)

Greg Guerin, Tom Saleeba, Samantha Munroe, Bernardo Blanco-Martin, Irene Martin-Fores, Andrew Tokmakoff

Maintainer: Greg Guerin <ggueri01@gmail.com>

References

Guerin, G. R., Sparrow, B., Tokmakoff, A., Smyth, A., Leitch, E., Baruch, Z., & Lowe, A. J. (2017). Opportunities for integrated ecological analysis across inland Australia with standardised data from Ausplots Rangelands. *PLoS ONE*, **12**, e0170137.

NVIS Technical Working Group (2017). Australian Vegetation Attribute Manual: National Vegetation Information System, Version 7.0. Department of the Environment and Energy, Canberra. Prep by Bolton, M.P., deLacey, C. and Bossard, K.B. (Eds)

Sparrow, B., Foulkes, J., Wardle, G., Leitch, E., Caddy-Retalic, S., van Leeuwen, S., Tokmakoff, A., Thurgate, N., Guerin, G.R. and Lowe, A.J. (2020). A vegetation and soil survey method for surveillance monitoring of rangeland environments. *Frontiers in Ecology and Evolution*, **8**, 157.

Tokmakoff, A., Sparrow, B., Turner, D., & Lowe, A. (2016). AusPlots Rangelands field data collection and publication: Infrastructure for ecological monitoring. *Future Generation Computer Systems*, **56**, 537-549.

White, A., Sparrow, B., Leitch, E., Foulkes, J., Flitton, R., Lowe, A. J., & Caddy-Retalic, S. (2012). AUSPLOTS rangelands survey protocols manual. University of Adelaide Press.

See Also

`get_ausplots`

Examples

```
#load package:
library(ausplotsR)
```

```
#get data for selected plots:
my.data <- get_ausplots(my.Plot_IDs=c("SATFLB0004", "QDAMGD0022", "NTASTU0002"),
veg.PI=FALSE, basal.wedge=TRUE, structural_summaries=TRUE)

#view module:
my.data$struct.summ

#get site info, veg vouchers and veg point intercept for all available plots:
## Not run: get_ausplots()

#get a species by sites table:
my.data <- get_ausplots(my.Plot_IDs=c("SATFLB0004", "QDAMGD0022", "NTASTU0002"))

species_table(my.data$veg.PI, m_kind="percent_cover", cover_type="PFC", species_name="SN")
```

`ausplots_visual` *Graphical illustration of sample TERN AusPlots data*

Description

Generates a set of graphical displays representing TERN AusPlots data based on geographic locations and vegetation attributes from the plot-based point intercepts (as generated by `get_ausplots` and other pre-processing functions).

Usage

```
ausplots_visual(my.ausplots.object = NULL, map = TRUE, map.attribute = TRUE,
fraction.pie = TRUE, growthform.pie = TRUE, cumulative.cover = TRUE,
whittaker = TRUE, outfile=NULL, max.plots = 5)

map_ausplots(my.ausplots.object)
```

Arguments

`my.ausplots.object`
: Optional (but required for `map_ausplots`) input raw data object as generated in a call from the `get_ausplots` function. The default graphical outputs require $site.info (for locations) and $veg.PI (for vegetation attributes) to be included in the AusPlots data extract.

`map`
: Logical, whether to include a basic map of site locations in the context of Australia.

`map.attribute`
: Logical, whether to include a map depicting site locations in Australia coded by cover of trees over 5 m high.

`fraction.pie`
: Logical, whether to include pie charts depicting `fractional_cover`.

`growthform.pie`
: Logical, whether to include pie charts depicting relative cover of plant growth forms.

`cumulative.cover`
: Logical, whether to include graphs of cumulative cover estimates by species as point intercepts are taken across each AusPlot.

`whittaker`
: Logical, whether to include Whittaker plots depicting species abundance distributions.

`outfile`
: Optional file path and name to which the output pdf file is written.

`max.plots`
: Maximum number of sites for which to display individual graphics panels. By default, `max.plots` sites are selected randomly. If you provide existing AusPlots data, `max.plots` sites are processed (in order) whenever `fractional.pie` or `cumulative.cover` are selected, because those calculations can be slow across a large number of plots.

Details

By default, extracts sample data, allowing the user to explore the dataset in a single call, or to easily visualise an extracted AusPlots dataset. Included in the default demonstration plots are maps of plot locations with and without coding by tree cover, and then a set of four panels for a smaller sample of AusPlots, depicting data from the main vegetation tables in the package, i.e., fractional cover, growth form cover, species cover and species relative abundance. This function is intended as a simple demonstration of the data available. Refer to TERN tutorials for many other ways to visualise and explore AusPlots.

Value

Plots are written to a pdf file to ensure consistent formatting, saved by default in the working directory.

Author(s)

Greg Guerin, Bernardo Blanco-Martin

References

Blanco-Martin, B. (2019) Tutorial: Understanding and using the 'ausplotsR' package and AusPlots data. Terrestrial Ecology Research Network. Version 2019.04.0, April 2019. https://github.com/ternaustralia/TERN-Data-Skills/

Guerin, G.R., Sparrow, B., Tokmakoff, A., Smyth, A., Leitch, E., Baruch, Z., Lowe, A.J. (2017) Opportunities for integrated ecological analysis across inland Australia with standardised data from AusPlots Rangelands. *PLoS ONE* **12**, e0170137.

Whittaker, R.H. (1965) Dominance and diversity in land plant communities: numerical relations of species express the importance of competition in community function and evolution. *Science*, **147**, 250-260.

See Also

`get_ausplots`

`fractional_cover`

growth_form_table

single_cover_value

species_table

Examples

```
## Not run:
#Default output
#Plot locations; visualisations for 5 random plots (including revisits):
ausplots_visual()

#Map tree cover only for all sites:

#Extract data first:
my.data <- get_ausplots(veg.vouchers=FALSE)

#Set maximum number of plots to all available in the veg.PI data:
ausplots_visual(my.data, map = FALSE, map.attribute = TRUE,
fraction.pie = FALSE, growthform.pie = FALSE, cumulative.cover = FALSE,
whittaker = FALSE, outfile = "AusPlots_treeCover.pdf",
max.plots=length(unique(my.data$veg.PI$site_location_name)))

## End(Not run)
```

---

basal_area *Basal area of tree trunks in TERN AusPlots*

Description

Calculates basal area (or number of basal wedge hits) for each plot, using the raw basal wedge data returned from `get_ausplots`.

Usage

```
basal_area(veg.basal, by.spp=FALSE, by.hits=FALSE,
species_name=c("SN","HD","GS"))
```

Arguments

veg.basal	Raw basal wedge data generated from get_ausplots.
by.spp	Set to TRUE to get values per individual species in plots rather than combined values per plot.
by.hits	Set to TRUE to return the number of individual basal wedge hits rather than basal area per se.
species_name	If by.spp=TRUE, set to "SN" to use "standardised_names" (the default), "HD" to use "herbarium_determination", and "GS" to use "genus_species". See details for explanation of each setting.

Details

Basal area is the cross-sectional area of tree trunks per unit area, and is measured at each plot using a basal wedge sweep. Measurement is by species at nine points. Basal area is averaged, while raw tree stem counts are also recorded.

`species_name` provides multiple options for calculating basal area if `by.spp=TRUE`. For example, if `species_name="HD"`, the function will use the "herbarium_determination" provided by state herbaria to identify unique species. "herbarium_determination" are made to the lowest possible taxonomic rank, which may be the family, genus, species, subspecies or variety. It includes incomplete identifications, dead or generic vegetation identifications (e.g. "Dead Tree/Shrub", "Annual Grass"), and "No ID" labels. If `species_name="SN"`, the default, the function will use the the "standardised_name" to identify unique species. Values are based on the `veg.PI$herbarium_determination` but have been edited to match the most commonly used or widely accepted scientific name according to "World Flora Online" (http://www.worldfloraonline.org/). "standardised_name" determinations are made to the lowest possible taxonomic rank, which may be the genus, species, subspecies or variety, but not family. It excludes generic vegetation identifications (e.g. "Dead Tree/Shrub", "Annual Grass") and "No ID" labels. If `species_name="GS"`, only the standardised genus species rank ("genus_species" in the veg.basal input table) is used to identify species. `species_name="GS"` maximises consistency in species comparisons between plots, but reduces the taxonomic detail and excludes records where only the genus was identified.

Value

Returns a dataframe with rows for plots (or species by plots) and basal area (m2/ha) or hit scores.

Author(s)

Greg R. Guerin, Samantha E.M. Munroe

See Also

`get_ausplots`

Examples

```
#get basal wedge data for selected plots:
my.data <- get_ausplots(my.Plot_IDs=c("SATFLB0004", "QDAMGD0022", "NTASTU0002"),
veg.vouchers=FALSE, site_info=FALSE, veg.PI=FALSE, basal.wedge=TRUE)

#calculate basal area:
basal_area(my.data$veg.basal)
```

`fractional_cover` *Fractional cover based on TERN AusPlots data.*

Description

Calculates fractional cover (i.e., the proportional cover of green vegetation, dead vegetation and bare substrate) based on plot-based point-intercept data from AusPlots (as generated by `get_ausplots`).

Usage

```
fractional_cover(veg.PI, ground_fractional="FALSE", in_canopy_sky="FALSE")
```

Arguments

`veg.PI`	The input raw point-intercept data as generated in the `get_ausplots` function.
`ground_fractional`	Set to "TRUE" to calculate fractional GROUND cover.
`in_canopy_sky`	Set to "TRUE" to include in green fraction, thereby calculating opaque canopy cover.

Details

Cover fractions are assigned according to the following:

'Green' or 'photosynthetic vegetation' is living vascular plant cover.

'Brown' or 'non-photosynthetic vegetation' is either vascular plant cover scored as 'dead' or substrate scored as litter, coarse woody debris or cryptogam (see below) that has no other veg cover.

'Bare' or 'bare ground' is substrate that is rock, outcrop, gravel or bare soil with no veg cover.

A height rule is applied so that coding to green/brown/bare of the uppermost substrate/vegetation stratum hit at a given point intercept location overrides the others. That is, a dead tree overrides a living shrub beneath and vice versa; substrate coding is overriden by any vegetation cover etc. This means for each of the (usually) 1010 intercepts, there is a single coding and percentage is the number of hits assigned to each fraction, divided by the total number of PIs taken (usually 1010 but can vary) times 100.

There is an option via argument 'ground_fractional' to calculate fractional ground cover - the same concept applied to only grasses (hummock, tussock, other); sedge; rush; forb; fern; and vine plant growth forms. Presently, cryptogam cover is excluded and included in the non-photosynthetic fraction.

'In canopy sky' is excluded by default (only the substrate is considered for those hits) and applies only to regular fractional cover (as trees are excluded in the green fraction for ground fractional cover by default).

Currently, cryptogam substrate is assigned to the non-photosynthetic fraction.

Occasionally substrate type was not collected ('NC') or could not be assigned to one of the above categories ('Unknwn'), in which case a percent cover will be returned under an 'NA' fraction if there was no veg cover above those points.

Value

Returns a data frame in which plots are rows, columns are fractions (bare, brown, green and NA) and values are percent cover.

Author(s)

Greg Guerin

See Also

get_ausplots

Examples

```
#get veg.PI data for selected plots:
my.data <- get_ausplots(my.Plot_IDs=c("SATFLB0004", "QDAMGD0022", "NTASTU0002"),
veg.vouchers=FALSE, site_info=FALSE)

#calculate fractional cover:
fractional_cover(my.data$veg.PI)
```

---

get_ausplots *Extract AusPlots data for sites, soils and vegetation*

Description

This function is the starting point for accessing data through the ausplotsR package.

Usage

```
get_ausplots(my.Plot_IDs="none", site_info=TRUE, structural_summaries=FALSE,
veg.vouchers=TRUE, veg.PI=TRUE, basal.wedge=FALSE, soil_subsites=FALSE,
soil_bulk_density=FALSE, soil_character=FALSE, bounding_box="none",
herbarium_determination_search=NULL, family_search=NULL,
standardised_name_search=NULL, dictionary=FALSE)
```

Arguments

my.Plot_IDs	Optional character vector of AusPlots plot IDs to request data for specific set of plots.
site_info	Whether site summary data are required (includes plot and visit details, landform data, geographic coordinates, notes etc). Default is to include.
structural_summaries	Whether site vegetation structural summaries are required.
veg.vouchers	Whether vegetation vouchers data are requested - contains a complete set of species records for the plot determined by a herbarium plus ID numbers for silica-dried tissue samples. Default is to include.
veg.PI	Whether point-intercept data are requested; includes data on substrate, plant species, growth form and height etc at each of (typically) 1010 points per plot. Default is to include.
basal.wedge	Whether basal wedge data raw hits are required to enable calculation of basal area by species by plot.
soil_subsites	Whether soil subsite information is required. This includes information on what soil and soil metagenomics samples were taken at nine locations across the plot and their identification barcode numbers.

soil_bulk_density
: Whether soil bulk density data are required.

soil_character
: Whether soil characterisation and sample ID data at 10 cm increments to a depth of 1 m are required.

bounding_box
: An additional optional spatial filter for selecting AusPlots based on a rectangular box, in the format of e.g. c(xmin, xmax, ymin, ymax). AusPlots location data are are in longlat, therefore x is the longitude and y is the latitude of the box/extent object. e.g., c(120, 140, -30, -10).

herbarium_determination_search
: Optional character string filter for selecting AusPlots based on the presence of a genus or species as recorded in the "herbarium_determination" column. See details for search term settings.

family_search
: Optional character string filter for selecting AusPlots based on the presence of a plant family as recorded in the 'family' column. Will be NA if no family identification was possible. See details for search term settings.

standardised_name_search
: Optional character string filter for selecting AusPlots based on the presence of a genus and/or species as recorded in the 'standardised_name' column. Will be NA if no genus rank (at minimum) was possible. See details for search term settings.

dictionary
: Whether the metadata variable dictionary is required. Provides a table that describes many of the variables and values in each data frame.

Details

By default, the function will extract and compile data from the vegetation vouchers and point intercept modules over all available plots and provide a basic site information table. Arguments allow the user to select sites by ID numbers, geographic bounding box, or species, and select which modules data are drawn from. Further spatial filtering of sites is possible by using the 'bioregion_name', 'state', and spatial coordinate fields in the site table.

The datasets returned by the function are used directly by further formatting and analysis functions in ausplotsR (such as `species_table` to generate a species by sites matrix for community analysis), making it simple to go from raw data extract to ecological analysis.

AusPlots surveys are identified by a plot ID and unique visit number, that are combined to identify a unique measurement for sites with repeat visits, identified in the extracted data tables as 'site_unique'.

"herbarium_determination_search", "family search", and "standardised_name_search" search terms are not case sensitive and do not require an exact taxonomic match (e.g. "Eucalyptus moderata", "Eucalyptus", and "euca" are all acceptable search terms). If `veg.vouch=TRUE`, veg.vouch will return a data frame that only includes voucher records that match the species or family search. If `veg.PI=TRUE`, veg.PI will return point intercept data for all plots where the species or family occurs. If `basal.wedge=TRUE`, veg.basal will return a dataframe that only includes the raw basal wedge data of records that match the species or family. For all other data.frames, get_ausplots will return data for all the plots where the species or family occurs.

`herbarium determinations` are provided by state herbaria and are the most commonly used scientific names in the given state. However, scientific names may differ between states and with international databases due to disagreements on taxonomy/nomenclature. Herbarium determinations are

made to the lowest possible taxonomic rank, which may be the family, genus, species, subspecies or variety. It also includes incomplete identifications, dead or generic vegetation identifications (e.g. "Dead Tree/Shrub", "Annual Grass"), and "No ID" labels.

`standardised_name_search` values are based on the `veg.PI$herbarium_determination` but have been edited to match the most commonly used or widely accepted scientific name according to "World Flora Online" (http://www.worldfloraonline.org/). This helps ensure scientific names will not differ between states and will be more consistent with global databases. `standardised_name_search` determinations are made to the lowest possible taxonomic rank, which may be the family, genus, species, subspecies or variety. It excludes dead or generic vegetation identifications (e.g. "Dead Tree/Shrub", "Annual Grass") and "No ID" labels. The genus, specific_epithet, infraspecific_rank, infraspecific_epithet and genus_species based on the standardised_name are listed as separate columns in veg.PI, veg.vouch, and veg.basal data tables. Phrase names supplied by herbaria that have not been formally published or described are not transfered to `standardised_name_search` determinations.

The metadata variable dictionary returned by `dictionary=T` may not include all of the the variables and values in each data frame, but will be expanded over time.

Value

Returns a list with following elements, depending on the AusPlots modules requested in the function call.

`$site.info` Data frame with basic site information including location.

`$struct.summ` Data frame with vegetation structural summaries for each plot.

`$veg.vouch` Data frame with rows for each voucher and information on species determinations and silica-dried tissue samples.

`$veg.PI` Data frame with individual point-intercept data.

`$veg.basal` Data frame with compiled raw basal wedge hit data.

`$soil.sub` Data frame with details of soil subsites within each plot including sample IDs.

`$soil.bulk` Data frame with raw bulk density data from each plot.

`$soil.char` Data frame with soil characterisation data from the 1 m pit at the SW corner of each plot.

`$metadata.dictionary` Data frame that lists and describes each variable and corresponding values in each data frame.

`$citation` Auto-generated citation for the data extracted. Please cite ausplotsR and the TERN AusPlots data you use.

Author(s)

Greg Guerin, Andrew Tokmakoff, Tom Saleeba, Samantha Munroe

See Also

species_table

Examples

```
#get data for selected plots:
my.data <- get_ausplots(my.Plot_IDs=c("SATFLB0004", "QDAMGD0022", "NTASTU0002"),
veg.PI=FALSE, basal.wedge=TRUE, structural_summaries=TRUE)

#View module:
my.data$struct.summ

#Get data citation and extraction date info:
my.data$citation

#Get site info, veg vouchers and veg point intercept for all available plots:
## Not run: get_ausplots()

#Get voucher records for "Eucalyptus moderata" using "herbarium_determination":
Eucalyptus_moderata <- get_ausplots(veg.PI=FALSE,
herbarium_determination_search="Eucalyptus moderata")
head(Eucalyptus_moderata$veg.vouch,10)

#Get veg voucher records for all species in the family "Myrtaceae"
Myrtaceae <- get_ausplots(veg.PI=FALSE, family_search="Myrtaceae")
head(Myrtaceae$veg.vouch,10)
```

---

growth_form_table *Plant growth form occurrence matrices for TERN AusPlots*

Description

Generates occurrence matrices for NVIS plant growth forms in plots as desired based on presence/absence, percent cover or species richness (i.e., the number of species assigned to a particular growth form). The input is a data frame of raw point intercept data from AusPlots generated using the get_ausplots function. Alternatively, cover by strata can be calculated.

Usage

```
growth_form_table(veg.PI, m_kind=c("PA", "percent_cover", "richness"),
cover_type=c("PFC", "OCC"), cumulative=TRUE, by_strata=FALSE,
species_name=c("SN","HD","GS"))
```

Arguments

veg.PI	The input raw point intercept data generated by get_ausplots
m_kind	The desired scoring method: binary (presence/absence; PA), percent cover or species richness.
cover_type	A choice between PFC: 'projected foliage cover' and OCC: 'opaque canopy cover' and only applies to percent_cover. If 'PFC', the default, is selected (suitable as an abundance or cover measure for most applications), hits scored as 'in

canopy sky' are removed, whereas they are retained as cover for that species for 'OCC' (suitable for application such as comparisons to remotely sensed cover).

cumulative — Logical. Should the cover of each growth form be calculated cumulatively across species (i.e., the sum of species covers in that growth form category; the default) or as absolute cover (percent of points where that growth form was hit)? Ignored except when `m_kind="percent_cover"`.

by_strata — Logical. If set to `TRUE`, cover is calculated by strata (as defined by growth forms). Ignored except when `m_kind="percent_cover"`.

species_name — Select "SN" to use "standardised_names" (the default), "HD" to use "herbarium_determination", and "GS" to use "genus_species". See details for an explanation of each setting.

Details

Probably the most useful data output from this function is the matrix of plant growth forms against sites with percent cover. This could be used in manova, ordination, classification etc. It also provides a multivariate structural and functional response variable.

`species_name` provides multiple options for calculating plant growth form cover. If `species_name="HD"`, the function will use the "herbarium_determination" provided by state herbaria to identify unique species. "herbarium_determination" are made to the lowest possible taxonomic rank, which may be the family, genus, species, subspecies or variety. It includes incomplete identifications, dead or generic vegetation identifications (e.g. "Dead Tree/Shrub", "Annual Grass"), and "No ID" labels. If `species_name="SN"`, the default, the function will use the the "standardised_name" to identify unique species. Values are based on the `veg.PI$herbarium_determination` but have been edited to match the most commonly used or widely accepted scientific name according to "World Flora Online" (http://www.worldfloraonline.org/). "standardised_name" determinations are made to the lowest possible taxonomic rank, which may be the genus, species, subspecies or variety, but not family. It excludes generic vegetation identifications (e.g. "Dead Tree/Shrub", "Annual Grass") and "No ID" labels. If `species_name="GS"`, only the standardised genus species rank ("genus_species" in the veg.PI input table) is used to identify species. `species_name="GS"` maximises consistency in species comparisons between plots, but reduces the taxonomic detail and excludes records where only the genus was identified.

Note sometimes the same species is recorded with different growth forms in a plot and therefore the rowSums of the occurrence matrix when m_kind is set to "richness" can be higher than the observed species richness because the same species can count towards the weights for multiple growth forms.

By default, cumulative percent cover (percent of hits out of total number of point intercepts) is returned, meaning multiple species of the same growth form hit at a point add to the score, which can therefore exceed 100 percent. If `cumulative=FALSE`, non-cumulative cover is returned, whereby only one hit is counted per growth form per point-intercept, with a maximum of 100.

If `by_strata=TRUE`, returns percent cover of three pre-defined strata: Upper, Mid and Lower, based on the crude assignment of growth forms such that Upper includes "Tree Mallee", "Tree/Palm", "Tree-fern", "Epiphyte"; Mid includes "Shrub Mallee", "Shrub", "Grass-tree", "Chenopod", "Heath-shrub"; and Lower includes "Tussock grass", "Forb", "Vine", "Hummock grass", "Fern", "Sedge", "Rush". The argument `cumulative` still applies for cover by strata, where the default (`TRUE`) is to calculate the cumulative cover of species in each stratum. Absolute cover (i.e., maximum value of 100) of strata can be calculated by setting `cumulative=FALSE`.

The returned occurrence matrix (plant growth forms against sites) can be converted to long format (with rows as individual occurrences of growth forms) using:

`my.matrix <-growth_form_table(my.veg.PI,m_kind="percent_cover",cover_type="PFC")`

`library(reshape2)`

`my.matrix <-as.data.frame(melt(as.matrix(my.matrix)))`

`my.matrix <-my.matrix[-which(my.matrix $value == 0),]`

The `round` function can be used on the returned matrix to estimate percent covers to the nearest percent if desired (i.e., with digits=0)

Value

Returns a data frame with plant growth forms as columns and sites as rows. Values are determined by argument m_kind. For 'PA', binary presence/absence (0/1) is returned; for 'richness', the numbers of species (integer) in growth form categories are returned; for 'percent_cover', percent cover from point-intercept is returned (see `Details`).

Author(s)

Greg R. Guerin, Samantha E.M. Munroe

References

NVIS Technical Working Group (2017) Australian Vegetation Attribute Manual: National Vegetation Information System, Version 7.0. Department of the Environment and Energy, Canberra. Prep by Bolton, M.P., deLacey, C. and Bossard, K.B. (Eds)

See Also

`get_ausplots`

`single_cover_value`

Examples

```
#get veg.PI data for selected plots:
my.data <- get_ausplots(my.Plot_IDs=c("SATFLB0004", "QDAMGD0022", "NTASTU0002"),
veg.vouchers=FALSE, site_info=FALSE)

#generate a percent cover occurrence matrix for plant growth forms:
growth_form_table(my.data$veg.PI, m_kind="percent_cover", cover_type="PFC",
cumulative=FALSE, species_name="SN")

#calculate cover of strata:
growth_form_table(my.data$veg.PI, m_kind="percent_cover",
cover_type="PFC", cumulative=FALSE, by_strata=TRUE, species_name="SN")
```

optim_species *Optimisation methods for plot selection using different diversity indices in order to maximise species accumulation*

Description

This function applies different optimisation methods to select a subset of plots that maximise species accumulation. Optimisation methods vary based on the desired biodiversity parameters chosen by the user. The function operates under the 'Maximum covering problem' framework, which seeks to identify the plots that will protect the maximum number of species in a specified number of plots. The user specifies the number of plots to select, and each optimiser selects the plots that represent the largest number of species for that given number of plots.

Usage

```
optim_species(speciesVsitesMatrix, n.plt=250, richness=TRUE, RRR=TRUE,
CWE=TRUE, shannon=TRUE, simpson=TRUE, simpson_beta=TRUE,
start="fixed", plot_name= NULL, frequent=TRUE, random=FALSE,
iterations=10, plot=TRUE, verbose=TRUE)
```

Arguments

`speciesVsitesMatrix`	dataframe or matrix, formatted as species (presence/absence or abundance) versus sites, where rows are sites and columns are species. See `Details` for more information on formatting.
`n.plt`	Number of plots, n, conforming the subset to be optimised.
`richness`	Species richness selected as optimiser. By default is TRUE.
`RRR`	Range rarity richness selected as optimiser. By default is TRUE.
`CWE`	Corrected Weight Endemism selected as optimiser. By default is TRUE.
`shannon`	Shannon-Wiener diversity index selected as optimiser. By default is TRUE.
`simpson`	Simpson diversity index selected as optimiser. By default is TRUE.
`simpson_beta`	Simpson dissimilarity selected as optimiser. By default is TRUE.
`start`	Choose under which condition the first plot for the simpson_beta optimiser is selected. The default, "fixed", uses the most speciose plot as the start seed. See details for additional options.
`plot_name`	Specific plot to start the optimisation when the user has selected start = "defined". By default, `plot_name = NULL`. If rownames are provided, the `plot_name` should be in character format and must match the relevant rowname in the input species matrix. If there are no rownames, `plot_name` can be numeric and will specify the row to use as the start seed.
`frequent`	See Details. By default is TRUE.

`iterations`	Number of random seed replications for `simpson_beta` if `frequent=TRUE` and also number of random replicates if `random=TRUE`.
`random`	Generation of a set of random species accumulations. By default is FALSE
`plot`	Whether or not to immediately plot the species accumulation curves for all the optimisers.
`verbose`	Logical, whether to print progress of iterations to the console.

Details

The input is a species versus sites matrix or dataframe. If a dataframe, the first column can list the site names. Site names must contain text, they cannot be numeric. Depending on the desired optimiser, the input data needs to be either species presence/absence or abundance. For richness, RRR, CWE, and simpson_beta optimisers, a presence/absence input matrix is required. For shannon and simpson optimisers, an abundance matrix is required. If the input data include abundance, the function automatically generates binary presence/absence data when required. TERN plot species occurrence matrices generated by the ausplotsR function `species_table` can be directly incorporated into optimiser function.

The `start` refers to the first plot used as the starting seed when using the simpson_beta optimiser. If "fixed", the plot with the greatest species richness is chosen as the start seed. If "random", a random plot is selected as the start seed. If "defined" the user assigns a specific starting plot as the start seed. Note that if `start = "defined"` then the `plot_name` argument must be provided.

`plot_name` must be provided when start = "defined" for the simpson_beta optimiser. If the input matrix or dataframe contains site names, then `plot_name` must match the rowname of the desired site to be used as the starting seed. If the input matrix does not include site names, then `plot_name` should specify the row number to be used as the starting seed.

`richness` refers to Species richness, which is the count of the number of species present in a given site. It is best used when the goal is to identify biodiversity hotspots.

`RRR` refers to Range rarity richness, which is a rarity-weighted richness calculated as the inverse of the number of sites in which a species occur. It is best used when the goal is to identify areas of high biodiversity and biological uniqueness.

`CWE` refers to Corrected Weight Endemism, which is calculated as range rarity richness (RRR) divided by species richness. It is best used when the goal is to identify centers of endemism highlighting range-restricted species.

`shannon` refers to the Shannon–Wiener diversity index, which combines species richness and the evenness or equitability by computing the species' relative abundances. The Shannon-Wiener diversity index assumes that all species are represented in a sample and that they are randomly sampled. The Shannon–Wiener index is defined as $H = -\sum_i p_i \log_b p_i$, where p_i is the proportional abundance of species i and b is the base of the logarithm. It is most popular to use natural logarithms, but some argue for base $b = 2$ (which makes sense, but no real difference).

`simpson` refers to the Simpson diversity index which combines species richness and the evenness or equitability by computing the species' relative abundances. The Simpson diversity index is a dominance index, giving more weight to common or dominant species. The Simpson diversity index is based on $D = \sum p_i^2$ and returns $1 - D$

`simpson_beta` refers to the Simpson dissimilarity, which is based on diversity partitioning, which separates species replacement (i.e. turnover) from species loss (i.e. nestedness). The Simpson

dissimilarity corresponds to the turnover component of the Sorensen dissimilarity. Thus, it is used to maximise species turnover.

By default, the function will extract and compile data from the top n selected plots (as specified by the user) based on all the different optimisers. The starting seed by default will be "fixed" which will correspond to the site with the highest value of species richness.

`frequent` refers to the most frequent sites that have been selected using the simpson_beta optimiser in a certain number of iterations that must be defined by the user. In order to do so, the starting seed for the simpson_beta optimiser must be "random". The result will display two different accumulation curves: one with the most frequent selected plots and another one with the mean and standard deviation of all the species accumulation curves obtained with a random starting seed for all the iterations

`random` refers to the species accumulation when the sites are selected randomly.

`plot` calls a function that allows plotting all the species accumulation curves obtained for each of the optimisers included in the optim_species function, see `plot_opt`.

Value

Returns a list containing, for each optimiser within the function, a species accumulation object (see `specaccum`, object as returned from vegan R package). The species accumulation refers to the cumulative curve or the number of species for a certain number of selected sites or individuals. Likewise, a list of the sites selected in order to maximise the value of the species accumulated using each optimiser will be returned. Additionally, the function will plot by default of the species cumulative curves obtained by the different optimisers.

Author(s)

Irene Martin-Fores, Samantha Munroe and Greg Guerin.

References

Albuquerque, F. & Beier, P. (2015) Rarity-weighted richness: a simple and reliable alternative to integer programming and heuristic algorithms for minimum set and maximum coverage problems in conservation planning. *PLoS ONE* **10**, e0119905.

Baselga, A. (2010) Multiplicative partition of true diversity yields independent alpha and beta components; additive partition does not. *Ecology* **91**, 1974-1981.

Baselga, A. (2012) The relationship between species replacement, dissimilarity derived from nestedness, and nestedness. *Global Ecology and Biogeography* **21**, 1223-1232.

Baselga, A. & Leprieur, F. (2015) Comparing methods to separate components of beta diversity. *Methods in Ecology and Evolution* **6**, 1069-1079.

Guerin, G.R. & Lowe, A.J. (2015) 'Sum of inverse range-sizes'(SIR), a biodiversity metric with many names and interpretations. *Biodiversity and conservation* **24**, 2877-2882.

Guerin, G.R., Ruokolainen, L. & Lowe, A.J. (2015). A georeferenced implementation of weighted endemism. *Methods in Ecology and Evolution* **6**, 845-852.

Jost, L. (2007). Partitioning diversity into independent alpha and beta components. *Ecology* **88**, 2427–2439.

Koleff, P., Gaston, K. J., & Lennon, J. J. (2003). Measuring beta diversity for presence-absence data. *Journal of Animal Ecology* **72**, 367-382.

Oksanen, J. et al. (2016) vegan: Community Ecology Package. R package version 2.4-3. Vienna: R Foundation for Statistical Computing.

See Also

plot_opt

specaccum

fisher.alpha

Examples

```
#example with dune database from vegan
library(vegan)
data(dune)

optim_species(dune, n.plt=15, frequent=FALSE)

#example with auplots database from ausplotsR
## Not run:
library(ausplotsR)

ausplotsdata <- get_ausplots(site_info=TRUE, veg.PI=TRUE, veg.vouchers=FALSE)

ausplotsPAdata <- species_table(ausplotsdata$veg.PI, m_kind="percent_cover",
cover_type="PFC", species_name="SN")

optim_species(ausplotsPAdata, n.plt=5, iterations= 5)

optim_species(ausplotsPAdata, n.plt=5, start="defined", plot=TRUE,
plot_name="WAANUL0001-56966", frequent=FALSE, random=TRUE, iterations=20)

## End(Not run)
```

---

plot_opt *Plot a set of species accumulation curves together*

Description

This function plots different species accumulation curves obtained through different optimisers in the optim_species function.

Usage

```
plot_opt(optim_result, choices=c("Richness", "RRR", "CWE", "Shannon",
"Simpson", "SimpsonBeta", "Frequent", "SimpsonBeta_randSeed", "Random"))
```

Arguments

optim_result
: Object returned from the optim_species function, which is a list of species accumulation objects (see specaccum) obtained with the different optimisers.

choices
: select the optimisers from which the species accumulations curves are going to be plotted. The choices can be "Richness", "RRR", "CWE", "Shannon", "Simpson", "SimpsonBeta", "Frequent", "SimpsonBeta_randSeed", "Random"

Details

Line colours are assigned randomly on each run.

Value

This function will return a plot of the species cumulative curves obtained by the different optimisers in the optim_species function.

Author(s)

Greg R. Guerin

See Also

optim_species

Examples

```
#example with dune database from vegan
library(vegan)
data(dune)

example1 <- optim_species(dune, n.plt=15, frequent=FALSE, plot=FALSE)

plot_opt(example1)
plot_opt(example1, choices=c("Richness", "SimpsonBeta"))

#example with auplots database from ausplotsR
## Not run:
library(ausplotsR)

ausplotsdata <- get_ausplots(site_info=TRUE, veg.PI=TRUE, veg.vouchers=FALSE)
ausplotsPAdata <- species_table(ausplotsdata$veg.PI, m_kind="PA",
species_name="SN")

example2 <- optim_species(ausplotsPAdata, n.plt=5, iterations=5, plot=FALSE)
plot_opt(example2)
plot_opt(example2,
choices=c("RRR", "SimpsonBeta", "Frequent", "SimpsonBeta_randSeed"))

## End(Not run)
```

single_cover_value — *Vegetation cover by height or growth form using TERN AusPlots data.*

Description

Calculates a single vegetation cover value per site based on the plot-based point-intercept data from AusPlots (as generated by `get_ausplots`). Cover can be subsetted to vegetation that has a specified minimum or maximum height and/or by plant growth forms, allowing user defined definitions of e.g. grass or tree cover. For example, by default, forest cover is calculated per plot for tree growth forms of 5 metres or higher.

Usage

```
single_cover_value(veg.PI, in_canopy_sky=FALSE,
by.growth_form=TRUE, min.height=5, max.height=NULL,
my.growth_forms=c("Tree/Palm", "Tree Mallee"))
```

Arguments

`veg.PI`	The input raw point intercept data ($veg.PI) as generated in the `get_ausplots` function.
`in_canopy_sky`	Logical, whether to include in canopy sky hits to calculate 'opaque canopy cover' instead of 'projected foliage cover'. Set to `TRUE` to calculate opaque canopy cover (e.g., suitable for some applications such as comparison to remotely sensed cover products).
`by.growth_form`	Logical, whether to subset by plant growth form. If set to FALSE then only height subsetting occurs. Set to TRUE to calculate cover subsetted to specified growth forms.
`min.height`	Numeric, sets the height in m below which PI hits are ignored. This argument is used to calculate e.g. cover of trees 5 m high or higher. Can be set to zero to ignore height and include any plant hit. If you set min.height to a negative number you will get a nonsensical output!
`max.height`	Numeric, sets the height in m above which PI hits are ignored. This argument can be used to calculate ground cover of desired growth forms, for example.
`my.growth_forms`	A user-supplied character vector specifying growth forms to include in the cover calculation. Defaults to trees. Applies only when `by.growth_form=TRUE`, otherwise this argument is ignored and only the height rule is applied. For allowable growth forms, see `unique(veg.PI$growth_form)`.

Details

All included point intercept hits are merged to give a single percent cover value with a maximum of 100. Cover partitioned by multiple plant growth forms can be calculated using `growth_form_table`, by species using `species_table` and fractional (green, brown or bare) cover using `fractional_cover`. Note, when min.height is set to zero and by.growth_form is set to FALSE, the output is nearly the

same as the green cover fraction returned from `fractional_cover`. The values can differ, however, because `fractional_cover` applies a height rule in which the highest intercept at a given point is taken, whereas `single_cover_value` finds any green cover, leading to differences, most obviously, for example, when dead trees overhang green understorey. For such general cover purposes, we recommend `fractional_cover`. `single_cover_value` is best suited to cover subset by height and growth form.

Value

Returns a data.frame in which plots are rows identified by a unique site and visit identifier ('site_unique') and the returned cover value is given in the percentCover column.

Author(s)

Greg Guerin

See Also

`get_ausplots`

`fractional_cover`

Examples

```
my.dat <- get_ausplots(bounding_box=c(140, 142, -20, -18), veg.vouchers=FALSE, site_info=FALSE)

#Default settings, where cover of trees 5 m or higher is calculated:
tree.cover <- single_cover_value(my.dat$veg.PI)
tree.cover

#Tussock grasses of any height (minimum height set to zero):
grass.cover <- single_cover_value(my.dat$veg.PI,
my.growth_forms=c("Tussock grass"), min.height=0)
grass.cover

#Any green veg at least 2 m in height:
veg.height.eg <- single_cover_value(my.dat$veg.PI, by.growth_form=FALSE,
min.height=2)
veg.height.eg

#Any green veg up to 1 m high:
ground.cover <- single_cover_value(my.dat$veg.PI, by.growth_form=FALSE,
min.height=0, max.height=1)
ground.cover
```

species_list *Species lists for AusPlots*

Description

Nice species lists based on the vegetation voucher module of Ausplots generated using the get_ausplots function.

Usage

```
species_list(veg.vouch, grouping=c("by_site", "by_visit", "collapse"),
species_name=c("SN","HD","GS"), strip_bryophytes=FALSE,
append_family=FALSE, writefile=FALSE, outfile="my_species_lists.txt")
```

Arguments

veg.vouch	The input raw plant voucher data generated by get_ausplots
grouping	One of c("by_site","by_visit","collapse"). Determines whether species names are pooled over all visits to each plot (site_location_name, the default), or gathered by individual site visits (site_unique). The final option 'collapse' returns a complete list of species over the plots provided.
species_name	Defaults to "SN" (standardised_name); set to "HD" to use "herbarium_determination", and "GS" to use standardised "genus_species" format. See details for an explanation of each setting.
strip_bryophytes	Logical. Switch to TRUE to exclude bryophyte records and only return vascular plants in Angiosperm (flowering plants), Gymnosperm (conifers) and Pteridophyte (ferns and allies) groups. Although there are a number of bryophyte records in Ausplots, they are not surveyed systematically.
append_family	Logical. If set to TRUE, names in the the returned species lists are appended with (and sorted by) family standardised name.
writefile	Logical. Whether or not to write the result to a text file.
outfile	Character denoting path/file/extension to attempt to write to if writefile=TRUE.

Details

The aim of the function is to provide simple, cleaned lists of species that have been recorded at various Ausplots sites, simplifying from the more detailed voucher and identification information in the $veg.vouch vegetation voucher module. The output is not designed for downstream analysis (which is perhaps better performed by manipulating the raw data tables) but as a rapid means of viewing recorded species.

species_name provides multiple options for how names are presented. If species_name="HD", the function will use the "herbarium_determination" provided by state herbaria to identify unique species. Herbarium determinations are made to the lowest possible taxonomic rank, which may be the family, genus, species, subspecies or variety. It includes incomplete identifications, dead or

generic vegetation identifications (e.g. "Dead Tree/Shrub", "Annual Grass"), and "No ID" labels. If species_name="SN", the default, the function will use the the "standardised_name" to identify unique species. Values are based on the veg.PI$herbarium_determination but have been edited to match the most commonly used or widely accepted scientific name according to "World Flora Online" (http://www.worldfloraonline.org/). "standardised_name" determinations are made to the lowest possible taxonomic rank, which may be the genus, species, subspecies or variety, but not family. It excludes generic vegetation identifications (e.g. "Dead Tree/Shrub", "Annual Grass") and "No ID" labels. If species_name="GS", only the standardised genus species rank ("genus_species" in the veg.vouch input table) is used to identify species. species_name="GS" maximises consistency in species comparisons between plots, but reduces the taxonomic detail and excludes records where only the genus was identified.

Value

Returns a list containing a character vector of species names for each plot/visit grouping. If grouping="collapse", a single character vector is returned. If writefile=TRUE, a text file is written to path outfile in the working directory.

Author(s)

Greg Guerin, Samantha Munroe

See Also

get_ausplots

species_table

Examples

```
#get veg.vouch data for selected plots:
my.data <- get_ausplots(site_info=FALSE, veg.PI=FALSE,
my.Plot_IDs=c("SAAFLB0005", "SAAFLB0006"))

#generate 'genus_species' lists by site:
species_list(my.data$veg.vouch, grouping="by_site",
species_name="GS", writefile=FALSE)

#generate a complete species list ordered by family:
species_list(my.data$veg.vouch, grouping="collapse",
species_name="SN", append_family=TRUE, writefile=FALSE)
```

---

species_table *Species occurrence matrices for AusPlots*

Description

This function takes a data frame of individual raw point-intercept hits from AusPlots generated using the get_ausplots function, and generates species occurrence matrices as desired based on presence/absence, cover, frequency or IVI.

Usage

```
species_table(veg.PI, m_kind=c("PA", "percent_cover", "freq", "IVI"),
cover_type=c("PFC", "OCC"), species_name=c("SN","HD","GS"),
strip_bryophytes=FALSE)
```

Arguments

- `veg.PI` The input raw point-intercept data generated by `get_ausplots`
- `m_kind` The desired species scoring method: PA: binary (presence/absence), percent cover, frequency (based on occurrences on the 10 point-intercept transects within plots) or IVI (combination of cover and frequency - see Details).
- `cover_type` A choice between PFC: 'projected foliage cover' and OCC: 'opaque canopy cover' and only applies to percent_cover and IVI. If 'PFC' is selected (the default), hits scored as 'in canopy sky' are removed, whereas they are retained as cover for that species for 'OCC'. 'PFC' may be more suitable for some applications such as comparison to remotely sensed cover products.
- `species_name` Set to "SN" to use "standardised_names" (the default), "HD" to use "herbarium_determination", and "GS" to use "genus_species" See details for an explanation of each setting.
- `strip_bryophytes` Logical. Switch to `TRUE` to exclude bryophyte records and only return vascular plants in Angiosperm (flowering plants), Gymnosperm (conifers) and Pteridophyte (ferns and allies) groups. Although there are a number of bryophyte records in Ausplots, they are not surveyed systematically.

Details

Species occurrence matrices are the basis for a wide range of downstream analyses in community ecology, such as classification, ordination, alpha and beta diversity, abundance distributions and many more. Many of these analyses can be performed in the vegan R package using the returned matrix.

IVI (Importance Value Index; Ellenberg & Mueller-Dombois 1974) is calculated as the sum of relative abundance (percent cover) and relative frequency (percent frequency of occurrence on the 10 transects used to gather the point-intercept data). It has a theoretical range of 0 - 200 and is calculated as follows:

$$(cover/totalcover) * 100 + (frequency/totalfrequency) * 100$$

`species_name` provides multiple options for calculating plant cover, presence or frequency. If `species_name="HD"`, the function will use the "herbarium_determination" provided by state herbaria to identify unique species. "herbarium_determination" are made to the lowest possible taxonomic rank, which may be the family, genus, species, subspecies or variety. It includes incomplete identifications, dead or generic vegetation identifications (e.g. "Dead Tree/Shrub", "Annual Grass"), and "No ID" labels. If `species_name="SN"`, the default, the function will use the the "standardised_name" to identify unique species. Values are based on the `veg.PI$herbarium_determination` but have been edited to match the most commonly used or widely accepted scientific name according to "World Flora Online" (http://www.worldfloraonline.org/). "standardised_name" determinations are made to the lowest possible taxonomic rank, which may be the genus, species, subspecies

or variety, but not family. It excludes generic vegetation identifications (e.g. "Dead Tree/Shrub", "Annual Grass") and "No ID" labels. If `species_name="GS"`, only the standardised genus species rank ("genus_species" in the veg.PI input table) is used to identify species. `species_name="GS"` maximises consistency in species comparisons between plots, but reduces the taxonomic detail and excludes records where only the genus was identified.

The returned occurrence matrix (species against sites) can be converted to 'long' format (with rows as species records) using:

```
my.matrix <-species_table(my.veg.PI,m_kind="PA",cover_type="PFC")
library(reshape2)
my.matrix <-as.data.frame(melt(as.matrix(my.matrix)))
my.matrix <-my.matrix[-which(my.matrix $value == 0),]
```

The `round` function can be used on the returned matrix to estimate percent covers to the nearest percent if desired (i.e., with digits=0)

Value

Returns a data frame with species as columns and sites as rows. Values are determined by argument `m_kind`.

Author(s)

Greg R. Guerin, Samantha E.M. Munroe

References

Ellenberg, D. & Mueller-Dombois, D. (1974) Aims and methods of vegetation ecology. Wiley, New York, NY.

See Also

`get_ausplots`

Examples

```
#get veg.PI data for selected plots:
my.data <- get_ausplots(my.Plot_IDs=c("SATFLB0004", "QDAMGD0022", "NTASTU0002"),
veg.vouchers=FALSE, site_info=FALSE)

#generate a percent cover occurrence matrix using "genus_species" species identification:
species_table(my.data$veg.PI, m_kind="percent_cover", cover_type="PFC",
species_name="GS")
```

Index

ausplots_visual, *4*, 5
ausplotsR (ausplotsR-package), 2
ausplotsR-package, 2

basal_area, *4*, 7

fisher.alpha, *19*
fractional_cover, *4–6*, 8, *22*

get_ausplots, *3–10*, 10, *13*, *15*, *21–26*
growth_form_table, *4*, *7*, 13

map_ausplots (ausplots_visual), 5

optim_species, *4*, 16, *19, 20*

plot_opt, *18, 19*, 19

round, *15*, *26*

single_cover_value, *4*, *7*, *15*, 21
specaccum, *18–20*
species_list, *4*, 23
species_table, *4*, *7*, *11, 12*, *17*, *24*, 24
